# Protective *PLCG2* variants associate with a delayed onset of Alzheimer’s disease among heterozygous *APOE* ε4 carriers

**DOI:** 10.1186/s13195-026-01957-1

**Published:** 2026-01-31

**Authors:** Heli Jeskanen, Sami Heikkinen, Inka Kervinen, Jenni Lehtisalo, Tiia Ngandu, Roosa-Maria Willman, Jessica Rosa, Dorit Hoffmann, FinnGen, Ville Leinonen, Annakaisa Haapasalo, Mari Takalo, Henna Martiskainen, Mikko Hiltunen

**Affiliations:** 1 Institute of Biomedicine, University of Eastern Finland, Kuopio, Finland; 2 Department of Public Health, Lifestyles and Living Environments Unit, Finnish Institute for Health and Welfare (THL), Helsinki, Finland; 3 Institute of Clinical Medicine/Neurology, University of Eastern Finland, Kuopio, Finland; 4 Division of Clinical Geriatrics, Center for Alzheimer Research, Care Sciences and Society (NVS), Karolinska Institutet, Stockholm, Sweden; 5 A. I. Virtanen Institute for Molecular Sciences, University of Eastern Finland, Kuopio, Finland; 6 Department of Neurosurgery, Kuopio University Hospital, Kuopio, Finland; 7 Institute of Clinical Medicine – Neurosurgery, University of Eastern Finland, Kuopio, Finland

**Keywords:** Alzheimer’s Disease, *PLCG2*-P522R, *PLCG2*, Ghrelin, *TREM2*, Onset age, *APOE*

## Abstract

**Background:**

The *PLCG2*-P522R variant, which encodes a mildly hyperactive form of the PLCγ2 enzyme, has been identified as a protective genetic factor against Alzheimer’s disease (AD). Many recently discovered AD-associated microglial risk genes converge on the TREM2-PLCγ2 signaling pathway, emphasizing the importance of characterizing this signaling pathway to uncover potential therapeutic targets and biomarkers. In this study, we investigated the effects of AD-associated *PLCG2* and *TREM2* variants, particularly in individuals carrying the *APOE* ε4 allele, and explored plasma biomarker profiles associated with these variants.

**Methods:**

Using genotype and clinical endpoint data from the FinnGen genomic research project, we conducted Kaplan–Meier survival analyses and Cox proportional hazards models to assess the ages of onset for AD, anxiety, and type 2 diabetes. The key findings were replicated in the UK Biobank datasets. Additionally, we assessed several metabolic and inflammatory plasma biomarkers in relation to *PLCG2* and *TREM2* variants among participants in the FINGER multi-domain lifestyle intervention cohort.

**Results:**

In FinnGen, both the *PLCG2*-P522R and *PLCG2*-3’UTR variants associated independently with a delayed age of AD onset, including among heterozygous *APOE* ε4 carriers. Also, carriers of the *PLCG2*-P522R variant showed significantly elevated plasma levels of ghrelin. Conversely, *APOE* ε4 carriers with the TREM2-R62H variant exhibited an earlier AD onset age. Similar trends for AD onset age were observed in the UK Biobank data.

**Conclusions:**

These findings indicate that protective *PLCG2* variants may mitigate *APOE* ε4-associated AD risk in the Finnish population. Moreover, the elevated plasma ghrelin levels observed in the carriers of the *PLCG2*-P522R variant suggest a potential connection between this metabolic hormone and beneficial anti-inflammatory or cognitive effects, although its specific role in AD remains uncertain. Collectively, our results highlight the need for additional studies to further elucidate the mechanisms and biomarkers through which protective *PLCG2* variants interact with *APOE* ε4.

**Supplementary Information:**

The online version contains supplementary material available at 10.1186/s13195-026-01957-1.

## Background

Genetic studies have identified a protective P522R variant in the *PLCG2* gene (*PLCG2*-P522R; rs72824905 C > G), which decreases the risk of Alzheimer’s disease (AD) and increases longevity [1–4]. *PLCG2*-P522R, which potentiates PLCγ2 function, mediates several beneficial effects on microglia function in in vivo and in vitro models [5–10]. Moreover, recent findings have revealed that neuronal downregulation of *PLCG2* impairs synaptic function and triggers AD-related alterations, suggesting that changes in PLCγ2 levels and subsequent functions also within neurons are significant in the context of AD [5].

PLCγ2 works downstream of triggering receptor expressed on myeloid cells 2 (TREM2) [11]. TREM2 is selectively expressed in microglia and it plays a key role in modulating the cell survival, phagocytosis, and inflammatory responses [3, 11, 12]. In *TREM2* gene, several AD associated risk increasing variants have been recognized, such as R47H and R62H [1, 13–15]. These variants impair TREM2 functions leading to reduced microglial activation and clearance of β-amyloid, which exacerbates the disease pathology [3, 11, 12]. It has been suggested that when compared to non-carriers, these variants do not differ in clinical presentation of the AD at baseline but they exhibit faster cognitive decline [13].

Here, we investigated the effects of the protective *PLCG2*-P522R and *PLCG2-*3’UTR (rs4243226 A > G) variants and the risk-increasing *TREM2*-R62H on the onset age of AD individually and in relation to apolipoprotein E ε4 (*APOE ε*4) allele in the large FinnGen cohort originating from Finland [16]. FinnGen is a biobank-scale cohort integrating genotype data from approximately 500,000 Finnish participants with longitudinal health information derived from national registries. The dataset includes imputed genome-wide variant data, clinical diagnoses coded using ICD systems, medication records, and demographic variables. Currently, FinnGen provides ~ 4,500 curated disease endpoints, systematically defined from registry data, which allow to perform phenotype-specific analyses across a wide spectrum of diseases, including AD. Furthermore, to identify potential biomarkers associated with the protective *PLCG2* variants and *TREM2*-R62H risk variant, we assessed the effects of the three variants on the plasma levels of common metabolic and inflammatory markers within the well-established FINGER intervention cohort [17]. Here, we show that the protective *PLCG2*-P522R variant associates with delayed AD onset age among carriers of one *APOE ε*4 allele as well as associates with increased plasma levels of ghrelin, which is known to exert anti-inflammatory effects, metabolic regulation, and cognitive enhancement [18–20]. Furthermore, we show that the *PLCG2*-3’UTR variant delays the AD onset age among *APOE* ε4 carriers and in contrast, *TREM2*-R62H leads to earlier onset of AD.

## Materials and methods

### Participants

FinnGen phenotype information and clinical endpoints are based on national health registries, including hospital discharge, prescription medication purchase, and cancer registers. A list of endpoints can be found from https://www.finngen.fi/en/researchers/clinical-endpoints and they can be explored at https://risteys.finngen.fi/. We have utilized information from 493,563 individuals from the FinnGen data release R12 in the analyses. In G6_ALZHEIMER endpoint, 319,783 individuals older than 50 years with *APOE* ε3/3, ε3/4, and ε4/4 genotypes were selected to investigate the effects of *APOE ε*4 on the different variant carriers on the Kaplan-Meier curves and cox analysis. In other endpoints presented in this study, all carriers of the *PLCG2*-P522R, *PLCG2*-3’UTR, and *TREM2*-R62H variants regardless of age were included. The FinnGen Study combines genome data with digital health data based on national health registers [16]. FinnGen includes samples that have been collected from the Finnish biobanks as well as legacy samples, which are from previous research cohorts that has been transferred to the biobanks. The individuals in FinnGen have given written informed consent for biobank research based on the Finnish Biobank Act. Separate research cohorts that have been collected before the Finnish biobank Act (September 2013) and start of FinnGen (August 2017), have been collected based on study-specific consents and later transferred to the Finnish biobanks after approved by the Finnish Medicine Agency Fimea. LD metrics were done in the Sisu (v4.2) imputation panel used in FinnGen.

All FinnGen study subjects (Table 1.) have undergone genome-wide genotyping. Most of the subjects have been genotyped using FinnGen ThermoFisher Axiom custom array. Approximately 70,000 subjects have been genotyped with various Illumina GWAS arrays as they originate primarily from the National Institute of Health and Welfare biobank samples that were genotyped before FinnGen. Approximately 21 million variants per individual were imputed using Finnish whole-genome reference SISu v4.2 (approximately 8,700 individuals). All genotype data is in the human genome build GRCh38/hg38. The genotype probability threshold in FinnGen was set to 0.8 for all the genotypes investigated.

**Table 1:** Summary statistics of FinnGen cohort

FinnGen cohort	Total (n)
Study subjects	493,563
Mean age	60.8 ± 18.0
Females/Males	278,264/215,299
*APOE* ε4 carriers	146,826
*PLCG2*-P522R	1977
*PLCG2*-3’UTR	448,141
*TREM2*-R62H	7563
AD cases	13,770
Anxiety cases	24,328
T2D cases	54,678

*n* = total number

The FINGER cohort characteristics [21] and study design [22] have been described previously [17]. Individuals who have dementia or substantial cognitive impairment have been excluded. Here, we only utilized data from participants aged 60–78 years carrying *PLCG2-*P522R (n = 7), *PLCG2*-3’UTR (n = 63), or *TREM2*-R62H (n = 19), and non-carriers (n = 56), who did not carry any of the investigated variants (Table 2.). The sex distribution among non-carriers was similar to variant carriers. As the frequency of the protective G allele of the *PLCG2-*−3’UTR is high (0.7), all the *PLCG2*-P522R and the *TREM2*-R62H individuals identified in the FINGER cohort were also carriers of the *PLCG2*-3’UTR G allele. Plasma biomarkers at the baseline and CRP values during one-, five-, and seven-year follow-up were used in the analysis. Forty plasma biomarkers were measured using Bioplex human diabetes 10-plex (BioRad, 10010747) assay multiplexed with adiponectin and adipsin as well as cytokine targets: eotaxin, G-CSF, GM-CSF, IFN-2Rα, IFN-γ, IL-10, IL12(p40), IL-12(p70), IL-13, IL-15, IL-1β, IL-2, IL-3, IL-4, IL-5, IL-6, IL-7, IL-8, IP-10, MCP-1, MIP-1α, MIP-1β, IL-1ra, RANTES, TNF-α, TNF-β, VEGF, IL-1, and IL-17. FINGER [22] individuals were genotyped using Illumina Global Screening Assay and imputed with TOPMed reference panel as described previously by Bellenguez et al. [1].

**Table 2:** Summary statistics of FINGER cohort

(n)	*PLCG2*-P522R	*PLCG2*-3'UTR	*TREM2*-R62H	Non-carriers
Carrier	7	63	19	56
Mean age	69.5 ± 4.2	68.5 ± 4.8	69.3 ± 5.3	70.0 ± 4.8
Sex (f/m)	5/2	27/36	5/14	27/28
*APOE* ε4 carriers	3	20	5	18

f = female, m = male

The UK Biobank participants, data, and endpoint definitions are described in Supplementary Methods and Supplementary Table 3.

### FinnGen endpoints

Endpoint information can be found in the FinnGen and FinRegistery data portal Risteys (https://risteys.finngen.fi/, read 16.09.2025):

AD was defined as a diagnosis in the hospital discharge or cause of death registries with the ICD codes G30 (ICD-10) or 3310 (ICD-9). The remaining individuals were considered as controls. The mean age of the cases was 79.7 ± 7.8 years and controls 60.2 ± 17.8 years.

T2D was defined as a diagnosis in the hospital discharge or cause of death registries with the ICD codes E11 (ICD-10) or 250.A (ICD-9). The remaining individuals were considered as controls. Individuals with pancreatitis were removed from both cases and controls. The mean age of the cases was 63.6 ± 13.8 years and controls 58.6 ± 18.3 years.

Anxiety was defined as a diagnosis in the hospital discharge or cause of death registries with the ICD codes F41.2, F41.3, F41.8, F41.9 (ICD-10), 3000 A (ICD-9), or 3000 (ICD-8). The remaining individuals, excluding those with neurotic, stress-related and somatoform disorders were considered as controls. The mean age of the cases was 38.1 ± 17.0 years and controls 61.8 ± 17.8 years.

### Statistical analysis

Kaplan-Meier curves were generated using R and survminer package (v 0.5.0). Also, survival analyses were performed in R using survival package (v 3.8.3). Based on the time dependence of the tested variables, cox proportional hazard model or extended cox model was used. Model fit was tested using Schoenfeld residuals. Separate *APOE* ε3/3, ε3/4, and ε4/4 groups were created to analyze the effects of *APOE ε*4 because the cox models nor the extended cox models proportional hazard assumption were met. *APOE* ε3/3 and *APOE* ε3/4 groups were analyzed using the cox model, including sex as covariate. *APOE* ε4/4 group was further divided into females and males, and the groups were then separately analyzed using the cox model. The dose-dependent effects of the variants were investigated by comparing the HRs of the different groups. Differences in BMI between genotypes were tested using Two-Way ANOVA and Tukey’s post hoc test using R (4.5.0). Regional association plot for the AD endpoint was created using R (4.5.0) package topr (v 2.0.2) [23] and FinnGen AD endpoint GWAS summary statistics using genome wide significance threshold p = 5 × 10^–8^. Replication of the FinnGen AD onset age and cox regression model analyses were done in UK Biobank, which are described in Supplementary Methods.

Two-way ANOVA for biomarker data was performed in GraphPad Prism (v 10.4.2.633). Outliers from the data were identified using ROUT (Q = 1%) and excluded from the analysis and figures. Bonferroni correction was applied for the p-values to correct for multiple testing. Multiple regression analyses of ghrelin, leptin, and visfatin were done in SPSS (v 29.0.0.0) adjusting for age, sex, and *APOE ε*4 carrier status. CRP levels > 10 were considered as a sign of acute inflammation and were excluded. Differences in the CRP levels over time were tested using Linear Mixed effects model using lme4 (v 1.1–37) and lmerTest (v 3.1–3) packages in R (4.4.1). Data are presented as mean ± standard deviation (SD) or standard error of the mean (SEM).

## Results

### Protective *PLCG2* variants exhibit minimal linkage disequilibrium

To explore the role of *PLCG2* variants in FinnGen in detail, we first assessed the risk effects of the well-established rare *PLCG2*-P522R variant (rs72824905 C > G) (Table 1), which has previously been shown to confer significant protection against AD [1, 3]. Carriership of *PLCG2*-P522R showed the expected protective effect against AD in FinnGen (OR = 0.55, 95% CI: 0.40–0.77, p = 3.83 × 10^–4^). Furthermore, we identified a common variant in the 3’UTR of *PLCG2* (rs4243226 A > G, frequency for G allele: 0.7), which was associated with a significantly decreased risk of AD (OR = 0.92, 95% CI: 0.90–0.95, p = 2.55 × 10^–8^). The *PLCG2*-3’UTR variant was the only *PLCG2* variant in FinnGen to show a significant genome-wide association with AD for which reason, it was selected for further analysis (Supp. Figure 1A-B). Linkage disequilibrium (LD) metrics revealed r^2^ and D’ values of 0.0004 and 0.85, indicating a minimal correlation between the *PLCG2-*P522R and *PLCG2*-3’UTR variants despite the high degree of allelic co-segregation. The previously reported AD-associated protective *PLCG2*-5’UTR variant (rs12446759) [1], which was not in LD with *PLCG2*-P522R or *PLCG2*-3’UTR variants (r^2^ and D’ values < 0.06), did not reach the genome-wide significance threshold in FinnGen (Supp. Figure 1A–B) and was therefore excluded from further analyses.

### *PLCG2* variants associate with delayed onset age of AD

**Fig. 1:**
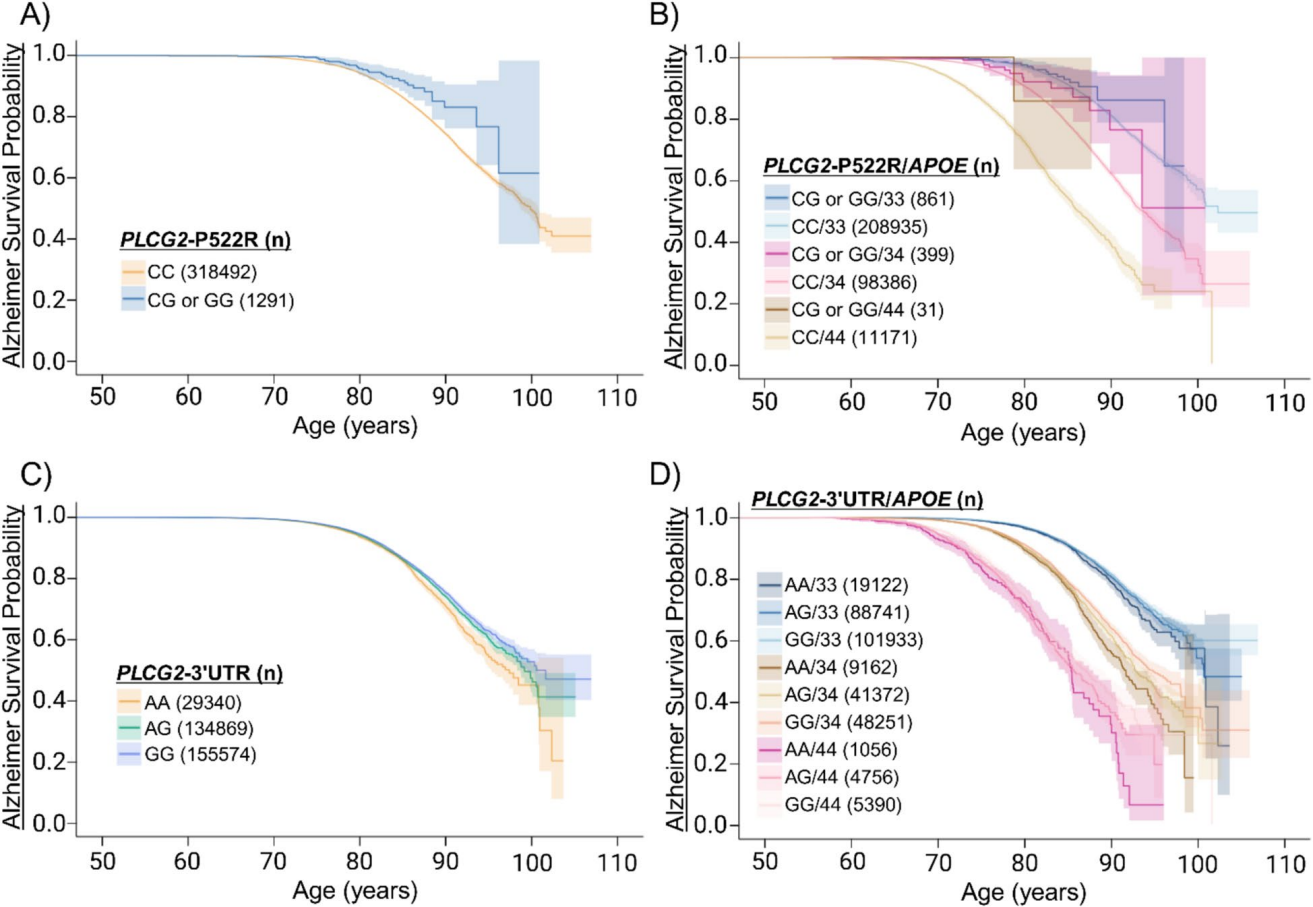
Protective *PLCG2* variants associate with delayed AD onset age. To investigate the impact of the *PLCG2*-P522R variant on AD onset, Kaplan–Meier curves on FinnGen endpoint data were utilized, with the focus on *APOE ε*3- and *APOE ε*4-carrying individuals > 50 years of age. The curves illustrate the AD-free time in years, starting from age 50 until the AD diagnosis or the end of follow-up for the controls. Shaded area indicates 95% confidence intervals. **A ***PLCG2*-P522R compared to non-carriers in FinnGen “ALZHEIMER” endpoint. **B ***PLCG2*-P522R carriers compared to non-carriers in FinnGen “ALZHEIMER” endpoint with *APOE ε*4 allele count. **C ***PLCG2*-3’UTR carriers compared to non-carriers in FinnGen “ALZHEIMER” endpoint. **D ***PLCG2*-3’UTR carriers compared to non-carriers in FinnGen “ALZHEIMER” endpoint with *APOE ε*4 allele count. *APOE*: ε3/ε3 = 33, ε3/ε4 = 34 and ε4/ε4 = 44

To study AD onset timing and risk, we used Kaplan-Meier survival curves to illustrate differences in onset age, while the Cox proportional hazards model estimated the risk of developing AD between the genotypes. Based on the survival analyses, *PLCG2*-P522R (CG or GG) significantly associated with delayed AD onset age as compared to non-carriers (CC) in FinnGen (Fig. 1A). Cox analysis revealed that *PLCG2*-P522R associated with a reduced risk by 42% of developing AD (HR = 0.58, 95% CI: 0.42–0.82, p = 0.00167). Additionally, male sex associated with an increased risk of AD (HR = 1.09, 95% CI: 1.05–1.13, p = 8.99 × 10^–7^). The *PLCG2*-P522R associated with a delayed onset of AD both in females and males (Supp. Figure 2A-B). In the *APOE* genotype*-*stratified analysis with sex as a covariate, *PLCG2*-P522R significantly associated with decreased risk of AD in the *APOE* ε3/ε4 group by 46% (HR = 0.54, 95% CI: 0.32–0.91, p = 0.022, Fig. 1B). The sex did not significantly affect the AD age of onset among the *APOE* ε3/ε4 carriers. However, male sex significantly associated with increased risk of having AD within in *APOE* ε3/ε3 carriers (HR = 1.20, 95% CI: 1.14–1.27, p = 3.7 × 10^–12^, Supp. Figure 2C-D). Within the *APOE* ε4/ε4 group, the *PLCG2*-P522R variant or sex did not significantly affect the AD onset age as compared to non-carrier AD patients (Fig. 1B and Supp. Figure 2C-D). Cox regression analysis of *PLCG2*-3’UTR revealed that this common variant (AG or GG) significantly associated with decreased risk of AD by 11–16% as compared to non-carriers (AA) (Fig. 1C). There were no significant differences between sexes with respect to *PLCG2*-3’UTR (Supp. Table 1, Supp. Figure 3A-B). According to *APOE* genotype-stratified analysis using sex as a covariate, the *PLCG2*-3’UTR variant did not significantly affect the AD onset age within the *APOE* ε3/ε3 group. Conversely, *PLCG2*-3’UTR moderately associated with increased AD onset age among homozygous *PLCG2*-3’UTR carriers as compared to non-carriers within the *APOE* ε3/ε4 group (HR = 0.83, 95% CI: 0.76–0.91, p = 4.32 × 10^–5^, Fig. 1D). Sex did not affect the AD onset age in the *APOE* ε3/ε4 group (Supp. Figure 3C-D). Interestingly, the homozygosity of *PLCG2*-3’UTR (GG) significantly associated with delayed AD onset age in the *APOE* ε4/ε4 carriers (HR = 0.87, 95% CI: 0.78–0.96, p = 0.0091, Fig. 1D). Furthermore, when sex effects were studied separately in *APOE* ε4/ε4 carriers, the *PLCG2*-3’UTR significantly associated with a protective effect only in female AD patients who were either heterozygous (HR = 0.74, 95% CI: 0.58–0.94, p = 0.016) or homozygous (HR = 0.65, 95% CI: 0.51–0.83, p = 0.00051) (Supp. Figure 3C-D) for the *PLCG2*-3’UTR variant. To assess the *PLCG2*-related findings beyond FinnGen, we conducted replication analyses in the UK Biobank. *PLCG2-*P522R and *PLCG2*-3'UTR variants were not significantly associated with delayed onset of AD, except among individuals homozygous for the *PLCG2*-3’UTR variant who also carried *APOE* ε3/ε4 (HR = 0.81, 95% CI: 0.66–0.98, p = 0.03) (Supp. Tables 4–7, Supp. Figure 5).

### GTEx analysis reveals *PLCG2*-3’UTR -associated shifts in *PLCG2* splice acceptor site usage

To investigate the potential functional role of the *PLCG2*−3’UTR variant, we used the Genotype-Tissue Expression (GTEx) database for eQTLs and splicing QTLs to indicate how genetic variants influence gene expression and splicing [24]. This analysis revealed that *PLCG2*−3’UTR acts as a splice QTL across multiple tissues, driving a shift in splice acceptor site usage: the G allele is strongly associated with increased utilization of the annotated acceptor site at chr16:81,937,758 (e.g., Whole Blood, p = 3.7 × 10⁻^11^) and decreased use of a cryptic, unannotated site at chr16:81,937,823 (e.g., Whole Blood, p = 9.7 × 10⁻^12^).

### *TREM2*-R62H variant associates with earlier AD onset age

**Fig. 2:**
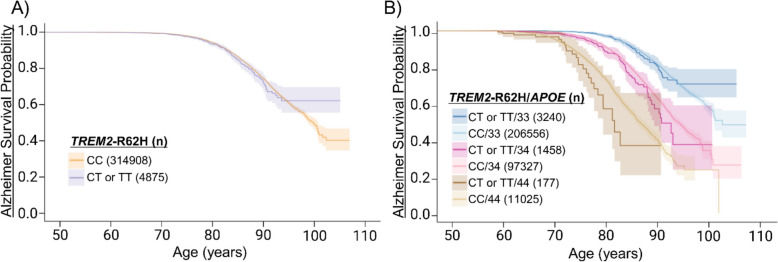
Risk increasing *TREM2*-R62H variant associates with decreased AD onset age. To investigate the impact of the *TREM2*-R62H variant on AD onset, Kaplan-Meier curves on FinnGen endpoint data were utilized, with the focus on *APOE ε*3- and *APOE ε*4-carrying individuals > 50 years of age. The curves illustrate the AD-free time in years, starting from age 50 until the AD diagnosis or the end of follow-up for the controls. Shaded area indicates 95% confidence intervals. **A ***TREM2*-R62H carriers compared to non-carriers and **B ***TREM2*-R62H carriers compared to non-carriers in FinnGen “ALZHEIMER” endpoint with *APOE ε*4 allele count. *APOE*: ε3/ε3 = 33, ε3/ε4 = 34 and ε4/ε4 = 44

Given that biologically PLCγ2 functions downstream of the TREM2 receptor, we also investigated the effects of the AD risk-increasing variant *TREM2*-R62H (rs143332484 C > T, OR = 1.21, 95% CI: 1.04–1.40, p = 1.3 × 10^–3^). The *TREM2*-R62H (AF = 0.0078) is more common in the Finnish population than the more widely known *TREM2*-R47H (AF = 0.00045) AD risk variant [15, 25]. No significant differences in the AD onset age were found between *TREM2*-R62H carriers (CT or TT) and non-carriers (CC) using the Cox model (Fig. 2A, Supp. Table 2). Also, no differences were observed between females and males (Supp. Figure 4A-B). In the *APOE* genotype-stratified analysis with sex as covariate, *TREM2*-R62H did not affect the AD onset age among the *APOE* ε3/ε3 carriers (Fig. 2B). Male sex significantly associated with increased risk of AD to a similar extent as observed in the *PLCG2*-P522R and *APOE* ε3/ε3-carrying individuals (Supp. Figure 4C-D). *TREM2*-R62H significantly associated with increased risk of AD by 23% (HR = 1.24, 95% CI: 1.01–1.52, p = 0.04, Fig. 2B) among the *APOE* ε3/ε4 group. Sex did not influence the AD onset age in *APOE* ε3/ε4 carriers. Among the *APOE* ε4/ε4 carriers, *TREM2*-R62H significantly associated with decreased the AD onset age only in males (HR = 1.80, 95% CI: 1.015–3.19, p = 0.04, Supp. Figure 4C-D). FinnGen analyses related to *TREM2*-R62H were also replicated in UK biobank. *TREM2*-R62H showed significant association with decreased AD onset age (HR = 1.44, 95% CI: 1.18–1.74, p = 0.00026, Supp. Table 8., Supp. Figure 6 A). Moreover, in the *APOE* stratified analysis *TREM2*-R62H significantly associated with earlier AD onset age among *APOE* ε3/ε3 (HR = 1.53, 95% CI: 1.11–2.11, p = 0.010) and ε3/4 (HR = 1.56, 95% CI: 1.20–2.03, p = 0.0011) carriers (Supp. Tables 9, Supp. Figure 6B).

### *PLCG2*-P522R variant does not increase longevity but protects against anxiety

**Fig. 3:**
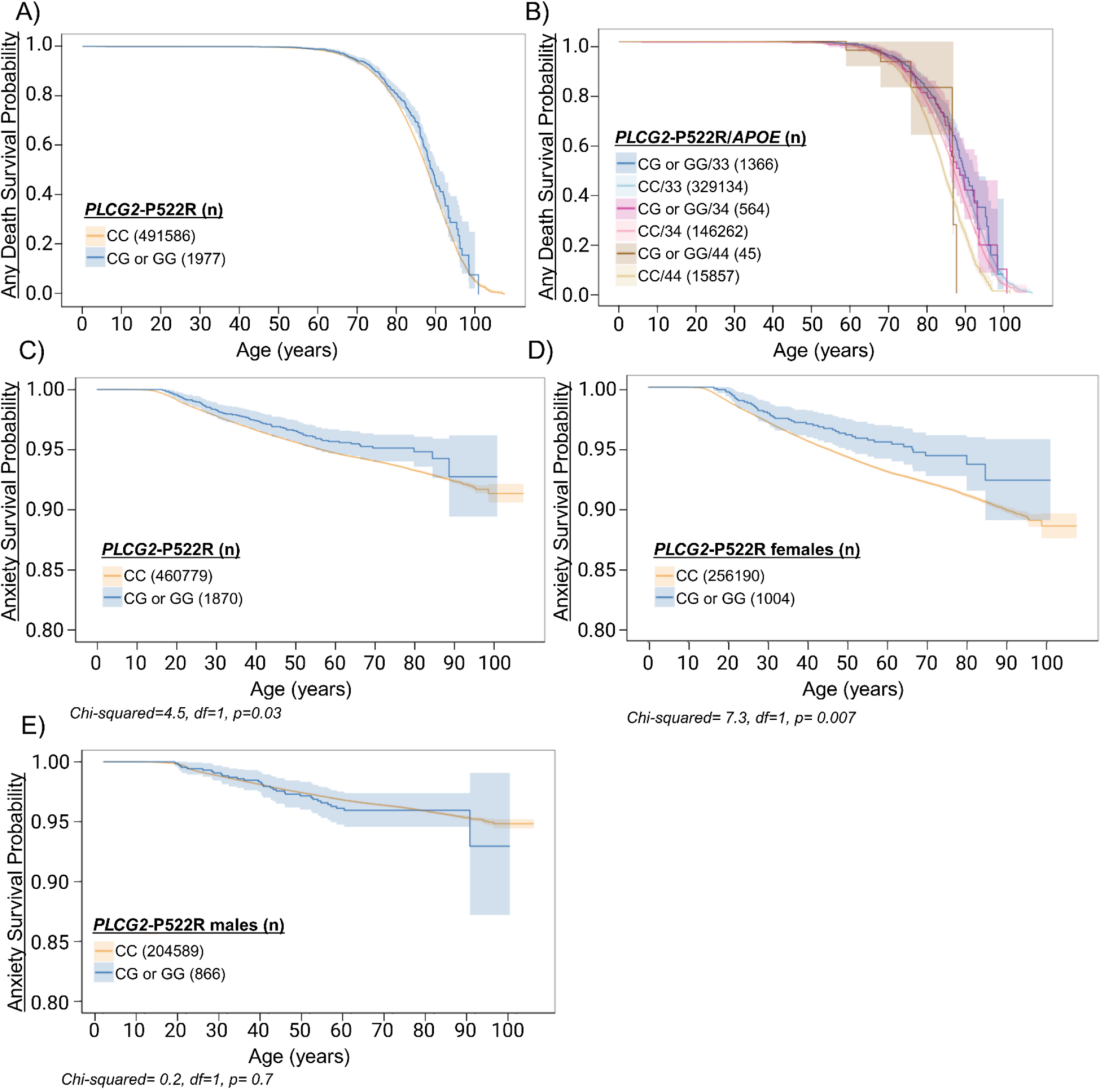
*PLCG2*-P522R associates with lower anxiety risk. To investigate the impact of the *PLCG2*-P522R variant on any death endpoint and anxiety onset, Kaplan-Meier curves on FinnGen endpoint data were utilized. The curves illustrate the death and anxiety-free time in years. The x-axis indicates the age at the first diagnosis for cases and the age at the end of follow-up for the controls. Shaded area indicates 95% confidence interval. **A ***PLCG2*-P522R carriers compared to non-carriers in “ANY DEATH” endpoint. **B ***PLCG2*-P522R carriers compared to non-carriers with *APOE ε*4 in “ANY DEATH” endpoint. **C** All carriers and non-carriers, **D **only females, and **E **only males in “ANXIETY” endpoint

As the *PLCG2*-P522R variant has been associated with longevity [1, 3], we investigated the FinnGen endpoint ‘Any Death’ to assess if the carriers lived longer than the non-carriers. However, we did not find any difference between the carriers and non-carriers (Fig. 3A). Also, there were no differences in *APOE* genotype-stratified analysis nor between sexes (Fig. 3B, Supp. Figure 7A-D).

Previously, some *PLCG2* hypermorphic mutations have been reported to associate with an increased risk of autoimmune diseases [26]. Thus, we examined whether homozygous *PLCG2*-P522R carriers exhibited signs of autoimmune diseases but found no significant associations. This observation highlights the beneficial nature of the *PLCG2*-P522R variant as opposed to strong hypermorphic mutations in *PLCG2* [27, 28]. In our recent study, we observed an anxiety phenotype in mice harboring the *PLCG2*-P522R [9]. Accordingly, we investigated if an association with anxiety can be detected in FinnGen in the individuals carrying the protective *PLCG2*-P522R. Unexpectedly, *PLCG2*-P522R significantly associated with delayed onset age of anxiety as compared to non-carriers (Fig. 3C). Notably, this effect was observed in female but not in male *PLCG2*-P522R carriers (Fig. 3C-E).

### *PLCG2*-P522R variant carriers show increased plasma levels of ghrelin

**Fig. 4:**
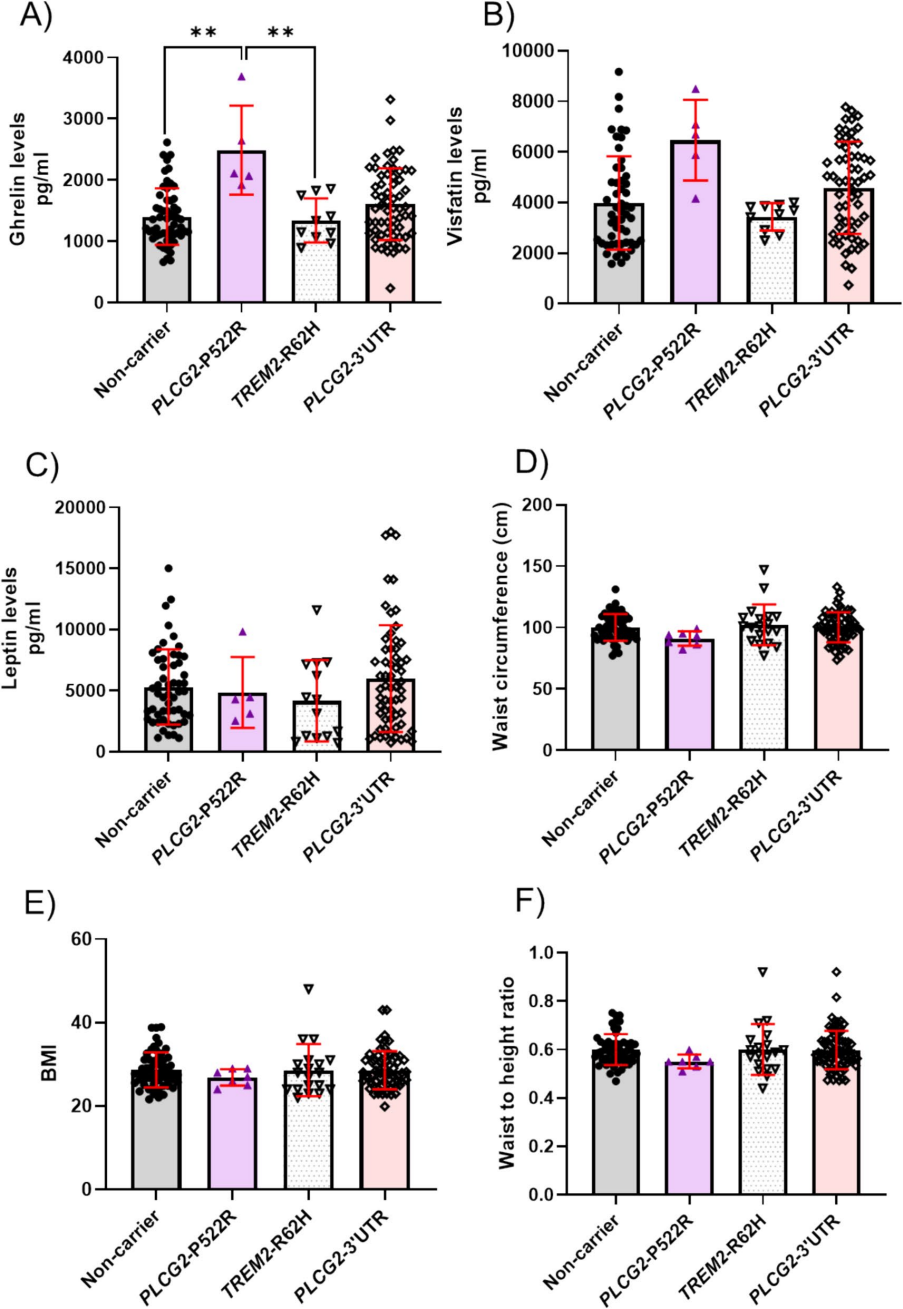
Protective *PLCG2*-P522R carriers show higher plasma ghrelin levels as compared to non-carriers and *TREM2*-R62H carriers. **A **Ghrelin, **B **Visfatin, and **C **Leptin levels in peripheral plasma samples from the FINGER cohort. **D **Waist circumference, **E **BMI, and **F **Waist-to-height ratio of FINGER individuals. n(control) = 53–56, n(P522R) = 5–7, n(R62H) = 10–18, and n(3’UTR) = 60–63. ANOVA, Tukey’s post hoc test, Bonferroni correction for multiple testing was applied. Mean ± SD. * < 0.05, ** < 0.01

**Fig. 5:**
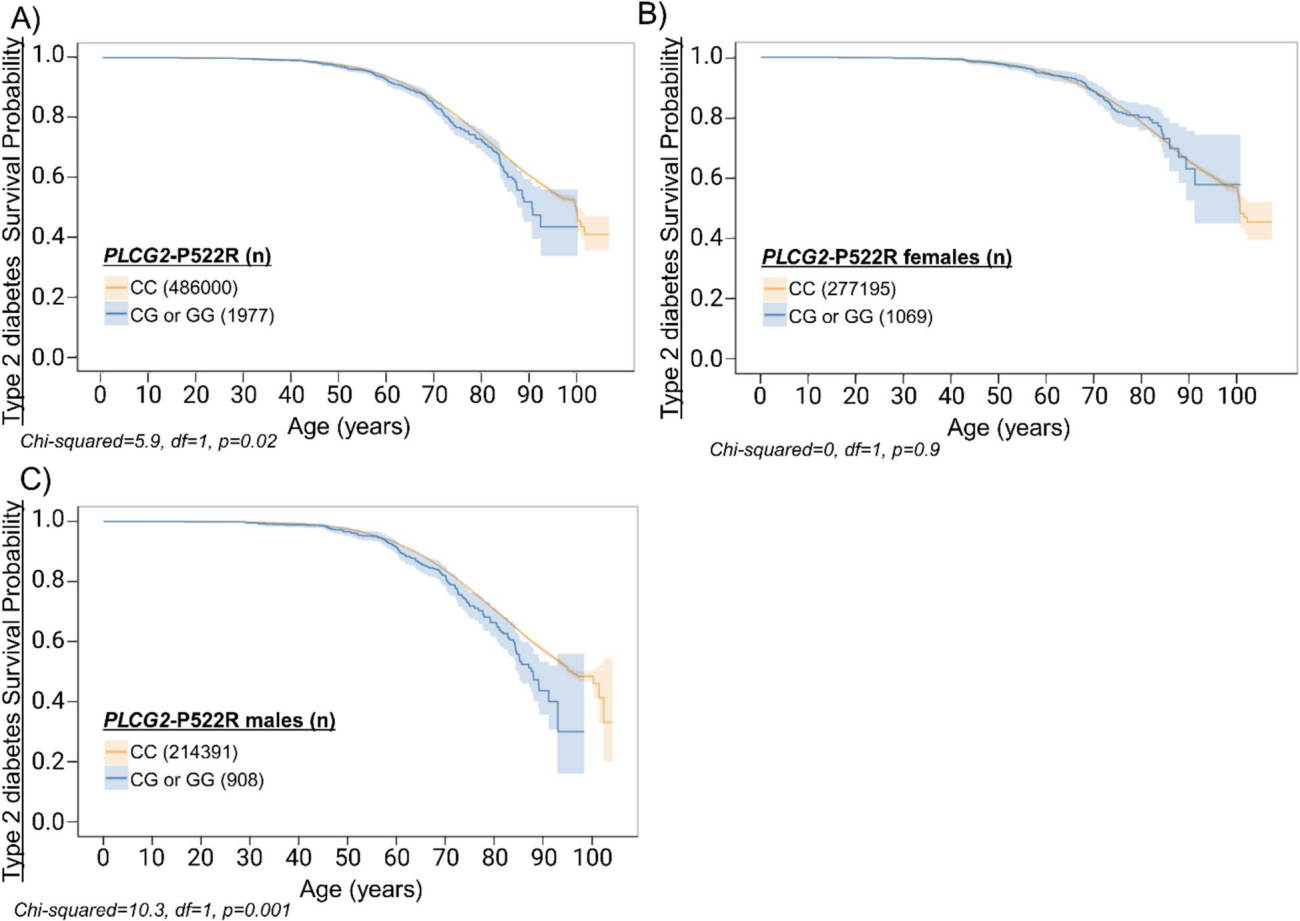
*PLCG2*-P522R associates with increased risk of type 2 diabetes in males. To investigate the impact of the *PLCG2*-P522R variant on T2D onset, Kaplan-Meier curves on FinnGen endpoint data were utilized. These curves illustrate the T2D-free time in years. The x-axis indicates the age at the first diagnosis for cases and the age at the end of follow-up for the controls. Shaded area indicates 95% confidence interval. **A ***PLCG2*-P522R FinnGen individuals as well as **B **only females and **C**) only males in T2D endpoint

To identify potential biomarkers associated with the *PLCG2* and *TREM2* variants, we analyzed 40 metabolism and inflammation-related markers in plasma samples of 145 FINGER cohort individuals [17, 21, 22] (Table 2). ANOVA analysis revealed a significant difference in the plasma levels of ghrelin among the groups (p = 0.0001 before Bonferroni correction and p = 0.004 after correction), and a nominally significant difference in visfatin (p = 0.0056 before Bonferroni correction and p = 0.224 after correction). Post-hoc comparison indicated significantly higher levels of ghrelin in *PLCG2*-P522R carriers as compared to non-carriers or *TREM2*-R62H variant carriers. There was no difference between *PLCG2*-P522R and *PLCG2*-3’UTR variant carriers (Fig. 4A-B). Since ghrelin and leptin jointly regulate energy metabolism, we also examined plasma leptin levels [29]. No significant differences were observed in the levels of leptin between *PLCG2*-P522R and non-carriers (Fig. 4C). *APOE* genotype nor sex affected the levels of ghrelin or visfatin (Supp. Figure 8A-E). However, males showed lower leptin levels than females (Supp. Figure 8 F). When ghrelin levels were tested using multilinear regression model adjusted by age, sex, and *APOE* genotype, the *PLCG2*-P522R was the only statistically significant effector (Supp. Table 10). In a similar analysis, there were no statistically significant differences in the levels of visfatin or leptin (Supp. Table 11–12). Due to the fact that ghrelin is known to increase appetite and affect peripheral metabolism [30], we investigated the waist circumference, BMI, and waist-to-height ratio of the *PLCG2* and *TREM2* variant carriers. No significant differences in these parameters between the genotypes were observed (Fig. 4D-F). Furthermore, levels of plasma C-reactive protein (CRP), an indicator of inflammation, remained unaffected at baseline and during seven-year follow-up measurements between the carriers and controls (Supp. Figure 9). Interestingly, when screening metabolism-associated endpoints in FinnGen, *PLCG2*-P522R was found to significantly associate with decreased onset age of type 2 diabetes (T2D), but only in males (Fig. 5A-C).

## Discussion

We and others have previously characterized the molecular mechanisms of the protective *PLCG2*-P522R variant in various models [6–10]. Building on this, we here examined the effects of the protective *PLCG2*-P522R variant on Finnish individuals using FinnGen [16], replicating the analysis in UK Biobank, and explored the potential plasma-based biomarkers in the FINGER cohort [17, 21, 22]. Alongside the *PLCG2*-P522R variant, we examined the newly identified common protective *PLCG2*-3’UTR variant and *TREM2*-R62H risk variant. Both *PLCG2* variants were associated with a delayed AD onset, also among carriers of one *APOE ε*4 allele, with the *PLCG2*-3’UTR variant showing a weaker, yet significant, effect. In contrast, *TREM2*-R62H variant was associated with an earlier AD onset among *APOE ε*4 carriers. Unfortunately, the low number of variant carriers limited the analysis of interaction between the *PLCG2* and *TREM2* variants. Furthermore, we observed increased plasma ghrelin levels in the *PLCG2*-P522R variant carriers compared to non-carriers.

In FinnGen, the protective *PLCG2*-P522R and *PLCG2*-3’UTR variants were associated with delayed AD onset age in *APOE* ε3/ε4, but not in *APOE* ε4/ε4 carriers, compared to non-carriers. In contrast, homozygosity for the *PLCG2*-3’UTR variant was associated with delayed AD onset age even among *APOE* ε4/ε4 carriers. Replication in the UK Biobank showed a similar, though non-significant, trend for the *PLCG2*-P522R variant, while the *PLCG2*-3’UTR variant remained significantly associated with delayed AD onset age among *APOE* ε3/ε4 carriers. Here, we show that *PLCG2*-P522R and *PLCG2*-3’UTR are not in LD, despite a high degree of allelic co-segregation. This finding suggests that the effects observed for these variants are independent. The dose–effect of harboring both protective variants in relation to *APOE* ε4 should be further explored to determine whether multiple protective *PLCG2* variants are associated with a greater delay in AD onset age. Another common variant, the *PLCG2*-5’UTR (rs12446759), has also been reported to be associated with the reduced risk of AD [1]. This variant is not in LD with *PLCG2*-P522R or *PLCG2*-3’UTR. However, the *PLCG2*-5’UTR was not included in our subsequent analysis because it did not reach genome-wide significance in the FinnGen. Collectively, these findings suggest that protective *PLCG2* variants can mitigate the *APOE ε*4-mediated risk of AD in individuals carrying one ε4 allele and highlight the independent protective effects of *PLCG2* variants.

*APOE ε*4 is known to disrupt lipid homeostasis and immune response and to cause mitochondrial dysfunction [31–34], while *PLCG2*-P522R variant has shown to exert opposing effects [7, 9]. In vivo studies have shown decreased and a more compact β-amyloid plaque area in *Plcg*2-P522R mouse brain, proposing a possible mechanism by which the *Plcg*2-P522R variant may render β-amyloid plaques less toxic to the surrounding neurons [9, 10]. Also, it was recently demonstrated that the *PLCG2*-P522R variant enhances immune responsiveness [35]. Conversely, loss-of-function variants in *PLCG2* have been shown to impair synaptic function [5]. These findings suggest that enhanced *PLCG2*-P522R-related mechanisms in both microglia and neurons may contribute to its protective effects. Although the *PLCG2*-3’UTR variant appears to have a similar effect on AD onset age as *PLCG2*-P522R, the molecular mechanism underlying its protective role remains unclear. From the mechanistic point of view, the *PLCG2*-3’UTR variant associates with cryptic splicing within exon 28 of *PLCG2* based on the GTEx database. The *PLCG2-*3’UTR A allele, but not the G allele, associates with the reduction of the full-length PLCγ2 levels by introducing a premature stop codon, but this mechanism requires experimental validation in future studies. It is important to note that the *PLCG2*-3’UTR variant is located several exons downstream of the implicated splice site, making a direct impact on splice acceptor usage elusive, despite the strong LD between *PLCG2*-3’UTR variant and cryptic splice region. Furthermore, splice QTL effects should be examined using single-cell RNA sequencing to determine whether the impact is restricted to peripheral tissues or could also involve brain cell types.

TREM2 is one of the key receptors modulating PLCγ2 activity [12, 36, 37]. Importantly, *TREM2* variants associate with an increased risk of AD [15, 25, 38–40]. TREM2 deficiency leads to impaired microglial response to β-amyloid plaques, accumulation of the lipid droplets, and increased β-amyloid load in the brain [11, 41]. Here, we investigated whether the *TREM2*-R62H variant exerts opposite effects on the onset age of AD and biomarker levels as compared to the *PLCG2*-P522R variant. We found that the *TREM2-*R62H variant decreases the onset age of AD, especially in the *APOE* ε4 carriers, both in FinnGen and UK Biobank. Given that *APOE* ε4 is the most well know genetic risk factor for AD, it is important to understand its complex interactions with other genetic variants, including *TREM2 and PLCG2*, when considering new therapies for AD [13, 14, 42]. In our present study, the *TREM2*-R62H variant significantly increases the risk of AD only when *APOE* ε4 is also present. This differs from Thomassen et al. [42] results, which showed that the *TREM2*-R62H variant significantly increases the risk of AD also in *APOE* ε3/3 carriers. Our findings for *TREM2*-R62H may reflect characteristics specific to FinnGen rather than the entire Finnish population, despite FinnGen representing ~ 10% of Finnish population. As FinnGen combines all major Finnish AD cohorts, replication in another Finnish cohort is not feasible. Likewise, the observed association of male sex with increased risk for certain variants may reflect FinnGen-specific effects. However, in line with our findings, also Thomassen et al. [42] reported that male *APOE ε*4/*ε*4 individuals with the *TREM2*-R62H variant had higher risk of having AD as compared to females, suggesting that *TREM2*-R62H has sex-dependent effect. Given that TREM2 activation may mitigate the harmful effects of *APOE* ε4, this underscores the importance of developing novel therapies targeting components of the TREM2 pathway, such as PLCγ2, which hold significant promise as potential treatment strategies for AD. This is especially needed among individuals with increased risk of AD owing to *APOE* ε4 background, which are not fully eligible for disease-modifying treatments, such as lecanemab [43]. Additionally, a deeper understanding of how sex influences AD risk across different genotypes is essential for developing effective therapeutic strategies.

Ghrelin, a neuroprotective hormone secreted by cells in the stomach, plays a key role in regulating the appetite and energy balance of the body [20, 29, 44]. It binds to growth hormone secretagogue receptor (GHSR), and it has been shown to protect neurons from β-amyloid-induced toxicity, reduce tau phosphorylation, and enhance synaptic plasticity [20, 44–46]. Despite its therapeutic potential, the role of ghrelin in AD pathology remains poorly understood. In our present study, carriers of the *PLCG2*-P522R variant exhibited higher plasma levels of ghrelin as compared to non-carriers and *TREM2*-R62H carriers, although the sample size was rather limited. This is an interesting finding, as elevated ghrelin levels are known to exert anti-inflammatory effects, metabolic regulation, and promote cognitive enhancement [20, 45, 47]. Importantly, BMI or waist circumference of the *PLCG2*-P522R variant carriers did not differ from the controls or carriers of the other investigated variants, although a slight trend towards a decrease was detected in the waist circumference among the *PLCG2*-P522R-carrying females as compared to *PLCG2*-3’UTR or *TREM2*-R62H variant carriers and non-carriers. It has been shown that ghrelin can pass through the blood–brain barrier, directly affecting brain cells [46, 48–50]. This raises the question of whether elevated ghrelin levels of *PLCG2*-P522R carriers contribute to neuroprotective processes. However, this finding needs to be validated in larger sample sets. In contrast to ghrelin, leptin levels remained unchanged in carriers of the protective *PLCG2*-P522R variant, suggesting that the hunger signal remains active. Supporting this observation, we previously demonstrated that aged knock-in mice homozygous for the *Plcg2*-P522R weighed less compared to their wild-type counterparts [9]. Here, we showed an earlier onset age of T2D in males but not in female *PLCG2*-P522R variant carriers. Additionally, onset of anxiety was delayed in females, suggesting sex-specific effects, consistent with prior findings [7]. While therapies aimed at increasing ghrelin levels may offer benefits in neurodegeneration, it should be considered that they could also disrupt the natural metabolic balance and increase the risk of severe adverse effects.

Interestingly, ghrelin has been observed to affect microglia polarization to an anti-inflammatory M2 type [51] and reduce the number of inflammatory M1 type microglia in cerebral ischemic injury [52]. However, direct evidence for ghrelin-mediated microglia modulation in the context of PLCγ2 signaling is lacking. Moreover, microglia lack the GHSR, the primary receptor for ghrelin [53–55]. Yet, ghrelin treatment has been shown to suppress inflammatory response and reduce reactive oxygen species production in microglial cells [53, 54]. Furthermore, ghrelin has also been shown to act as a mitochondrional mediator and enhance mitochondrial fitness upon inflammation in macrophages [56]. Whether similar mechanisms occur in microglia remains unclear. Hence, ghrelin could affect microglia functions indirectly by modulating other brain cell types that do express the GHSR, such as neurons [53, 54, 57] and astrocytes [58]. Moreover, the beneficial role of ghrelin in the central nervous system has been widely discussed, including enhanced cognition [48, 59–61]. However, there is still a lack of direct evidence on whether ghrelin or its active form acyl ghrelin can influence microglial metabolism or if the effects are mediated by the interaction of ghrelin with insulin signaling pathways or through neuronal pathways. Further studies are needed to clarify whether these biological processes are linked to the protective effects of *PLCG2*-P522R variant.

## Limitations

This study has certain limitations that should be considered when interpreting the results. First, the low frequency of the rare protective *PLCG2*-P522R variant reduces statistical power and prevents evaluation of its combined effects with rare *TREM2* risk variants, either independently or among *APOE* ε4 carriers. Second, because the *PLCG2* and *TREM2* analyses are based primarily on Finnish cohorts, the observed associations may reflect population-specific genetic architecture or environmental exposures unique to Finland. Factors, such as genetic drift and lifestyle could influence variant frequencies and effect sizes, which may explain why replication in the UK Biobank revealed similar trends but not identical results with *PLCG2* and *TREM2* variants. These discrepancies highlight the importance of validating findings in diverse populations to ensure generalizability. Third, reliance on Finnish biobank data introduces potential biases related to recruitment strategies, health record completeness, and phenotype definitions, even though clinically validated AD diagnoses were used. For example, the observed association of male sex with increased AD risk could partly reflect biobank selection bias compared to purely population-based recruitment. Finally, the absence of mechanistic data related to plasma ghrelin findings in *PLCG2*-P522R carriers add complexity to the interpretation of the obtained results, underscoring the need for experimental validation and replication in larger, preferably longitudinal cohorts.

## Conclusions

In conclusion, the obtained results strengthen the notion that the *PLCG2* protective variants may alleviate the effects of *APOE ε*4, the strongest genetic AD risk factor, in carriers of one *APOE ε*4 allele. Future studies should explore the mechanisms of how the protective *PLCG2* variants may mitigate *APOE ε*4-related impairments as it may offer new therapeutic avenues for a vast number of AD patients. Related to this, *PLCG2*-P522R carriers exhibited increased ghrelin levels. Although this finding requires confirmation in larger cohorts, ghrelin emerges as a promising candidate for modulating neurodegenerative processes.

## Supplementary Information

Supplementary Material 1. FinnGen Banner Sep2025.

Supplementary Material 2. Supplementary Figures

Supplementary Material 3. Supplementary Methods

Supplementary Material 4. Supplementary Tables

## Data Availability

Summary-level data from FinnGen Data Release 12 are publicly accessible via www.finngen.fi/en/access_results and https://r12.finngen.fi. Researchers may apply for access to individual-level genotype data through the Fingenious portal (https://site.fingenious.fi/en/), operated by the Finnish Biobank Cooperative FinBB (https://finbb.fi/en/). Applications for access to Finnish health register data are managed by Findata (https://findata.fi/en/data/). Endpoint information can be found in the FinnGen and FinRegistery data portal Risteys ([https://risteys.finngen.fi/], read 16.09.2025). FINGER dataset is owned by Finnish Institute for Health and Welfare. As it contains individual-level data that is not publicly accessible, researchers interested in using the dataset may request access by contacting the project coordinator, Tiia Ngandu (tiia.ngandu@thl.fi), or Senior Researcher Jenni Lehtisalo (jenni.lehtisalo@thl.fi).

## Abbreviations

AD: Alzheimer’s disease
PLCγ2: Phospholipase C γ2
TREM2: Triggering receptor expressed on myeloid cells 2
FINGER: Finnish Geriatric Intervention Study to Prevent Cognitive Impairment and Disability
ApoE: Apolipoprotein E
