# Supplementary material for: Protective PLCG2 variants associate with a delayed onset of Alzheimer’s disease among heterozygous APOE ε4 carriers
Source: Alzheimers Res Ther. 2026 Jan 31;18:53. doi: 10.1186/s13195-026-01957-1 (PMC12964913; doi:10.1186/s13195-026-01957-1)
Supplement: Supplementary file 2 — Supplementary Material 2. Supplementary Figures [file 13195_2026_1957_MOESM2_ESM.pdf]

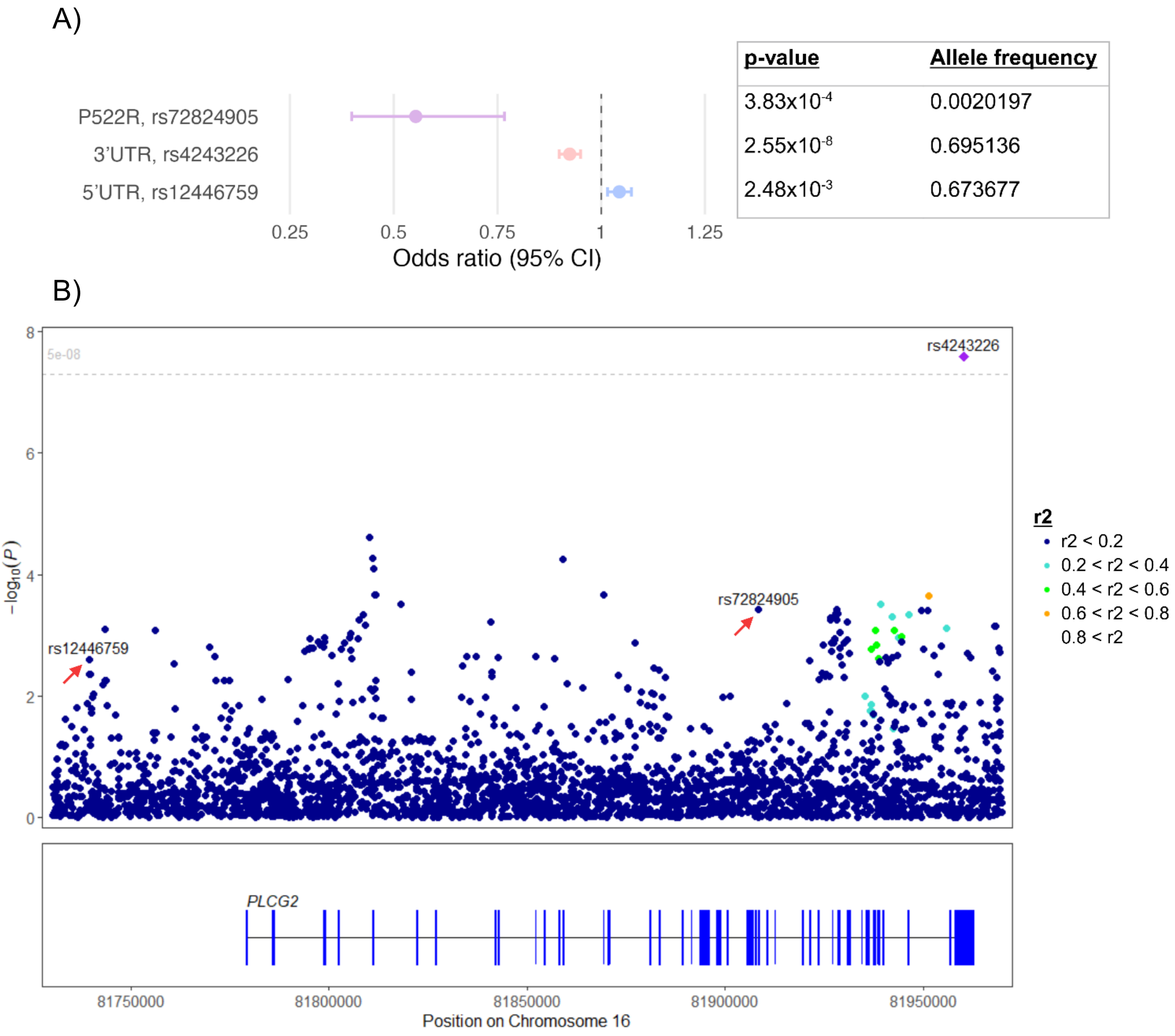

p-value	Allele frequency
$3.83x10^{-4}$	0.0020197
$2.55x10^{-8}$	0.695136
$2.48x10^{-3}$	0.673677

Supplementary Figure 1. Forest and regional association plots of *PLCG2*-P522R (rs72824905), *PLCG2*-3'UTR (rs4243226), and *PLCG2*-5'UTR (rs12446759) variants. A) Forest plot of odds ratios with 95% confidence intervals (CI), p-values and allele frequency in "ALZHEIMER" endpoint derived from FinnGen. B) Regional association plot of *PLCG2* gene locus showing genome-wide significance threshold in grey dotted line (equivalent to $p=5x10^{-8}$). The y-axis presents negative log10-transformed p-values for the endpoint "ALZHEIMER" from FinnGen summary statistics. The x-axis presents the chromosomal position (base pairs). Each dot

represents an individual variant, and the dot color represents LD with the lead variant (purple diamond). Non-significant variants *PLCG2*-P522R (rs72824905) and *PLCG2*-5'UTR (rs12446759) have been marked with red arrow.

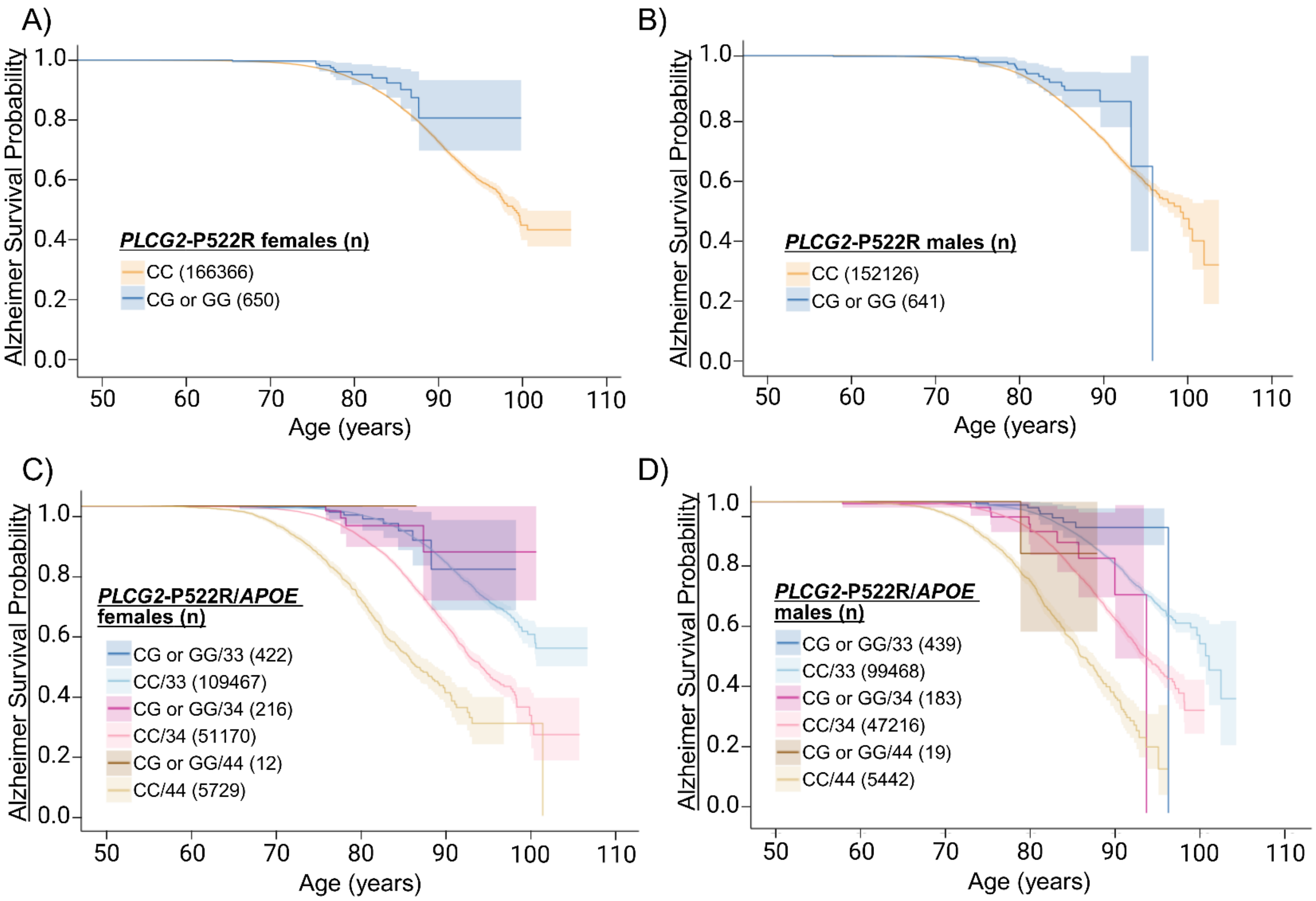

Supplementary Figure 2. *PLCG2*-P522R variant associates with delayed AD onset age. To investigate the impact of the variants on AD onset, Kaplan-Meier curves on FinnGen endpoint data were utilized, with the focus on *APOE* ε3- and *APOE* ε4-carrying individuals >50 years of age. The curves illustrate the AD-free time in years, starting from age 50 until AD diagnosis or the end of follow-up for the controls. Shaded area indicates 95% confidence interval. A) *PLCG2*-P522R females and B) males and as well as C) females and D) males with *APOE* ε4 allele count. *APOE*: ε3/ε3=33, ε3/ε4=34 and ε4/ε4=44.

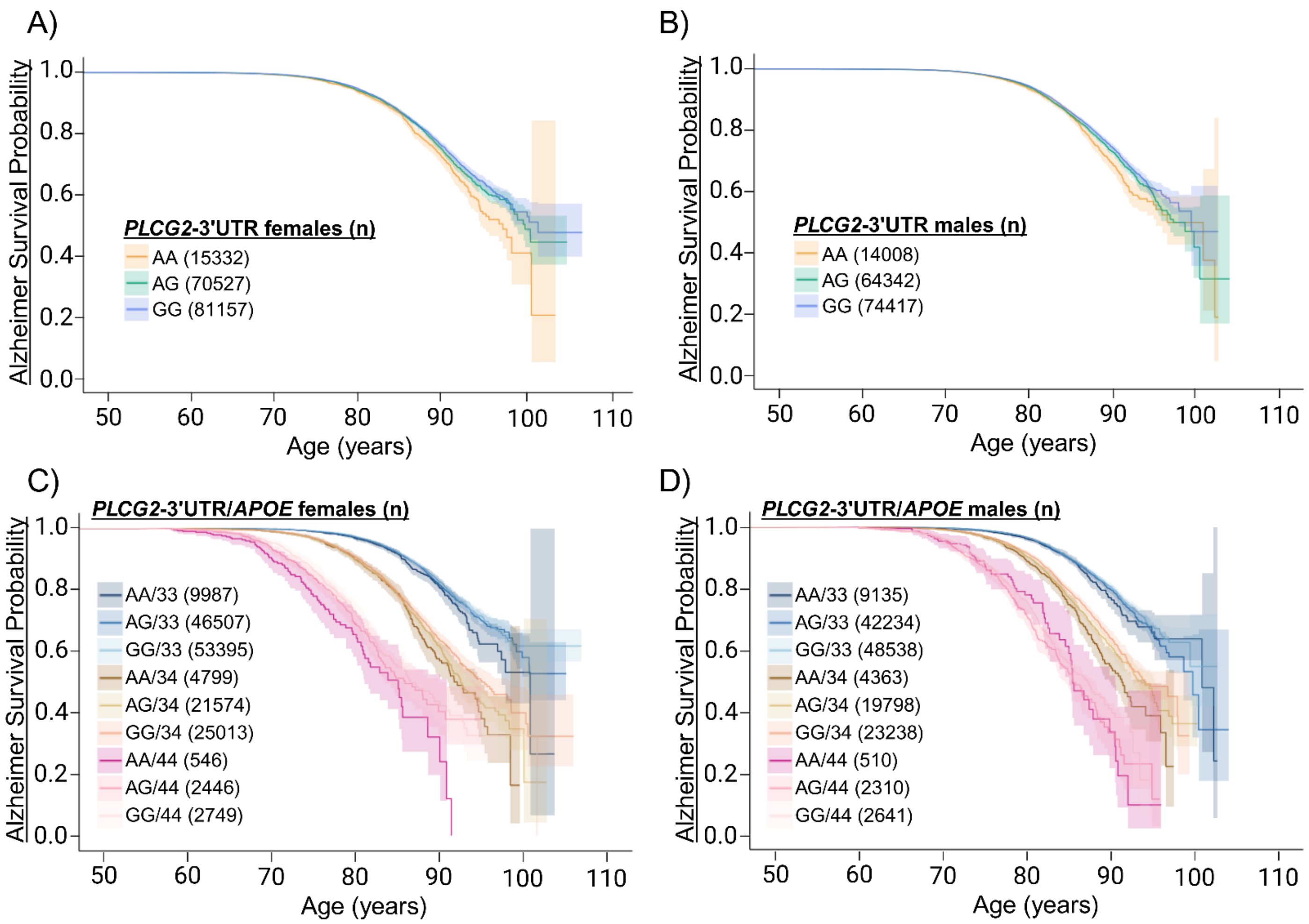

Supplementary Figure 3. *PLCG2*-3’UTR variant associates with delayed AD onset age. To investigate the impact of the variant on AD onset, Kaplan-Meier curves on FinnGen endpoint data were utilized, with the focus on *APOE ε*3- and *APOE ε*4-carrying individuals >50 years of age. The curves illustrate the AD-free time in years, starting from age 50 until AD diagnosis or the end of follow-up for the controls. Shaded area indicates 95% confidence interval. A) *PLCG2*-3’UTR females and B) males as well as C) females and D) males with *APOE ε*4 allele count. *APOE*: ε3/ε3=33, ε3/ε4=34 and ε4/ε4=44.

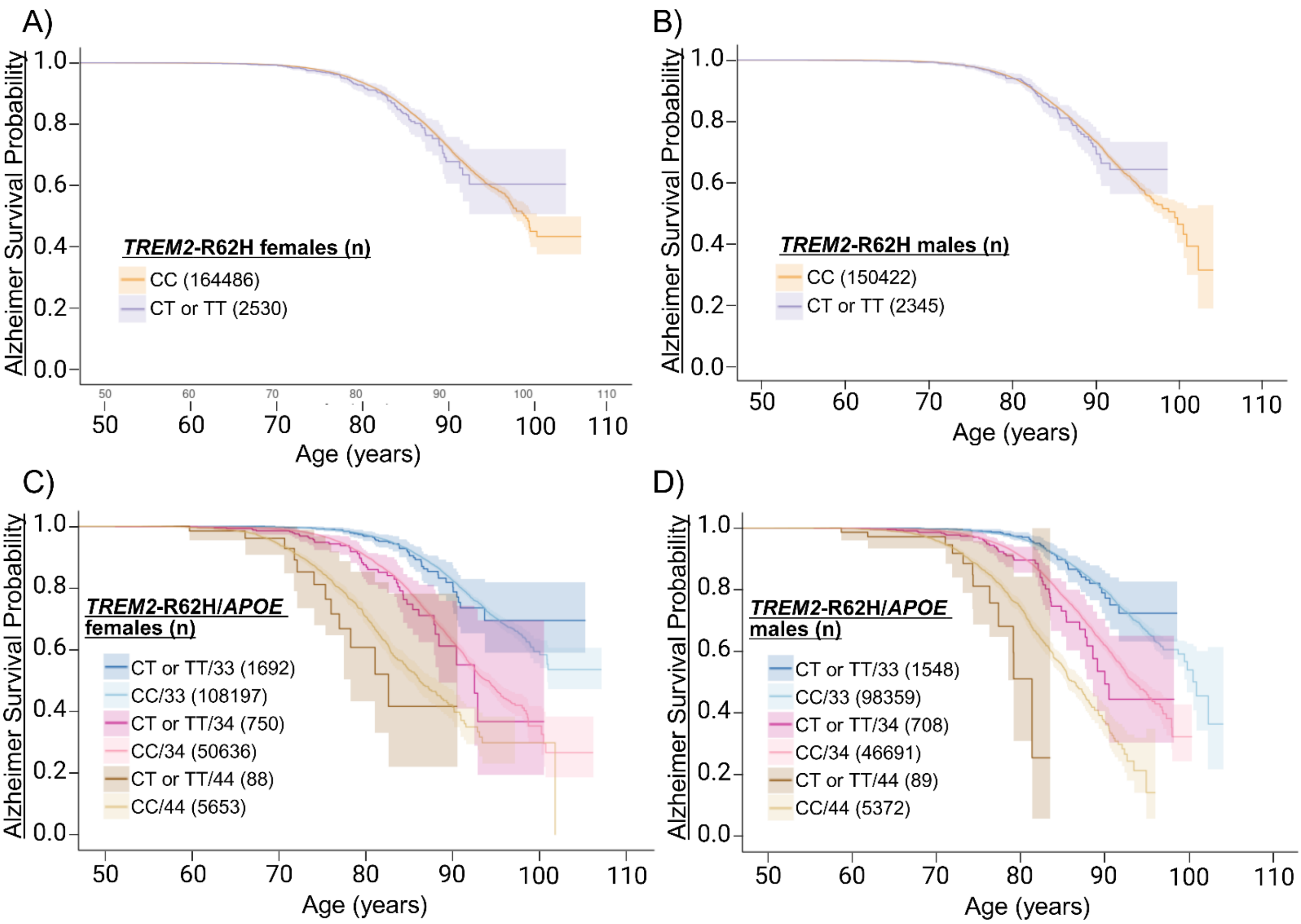

Supplementary Figure 4. *TREM2*-R62H associates with decreased AD onset age of *APOE ε*4 carriers. To investigate the impact of the *TREM2*-R62H variant on AD onset, Kaplan-Meier curves on FinnGen endpoint data were utilized, with the focus on *APOE ε*3- and *APOE ε*4-carrying individuals >50 years of age. The curves illustrate the AD-free time in years, starting from age 50 until AD diagnosis or the end of follow-up for the controls. Shaded area indicates 95% confidence interval. A) *TREM2*-R62H females, B) males as well as C) females and D) males with *APOE ε*4 allele count in "ALZHEIMER" endpoint. *APOE*: ε3/ε3=33, ε3/ε4=34 and ε4/ε4=44.

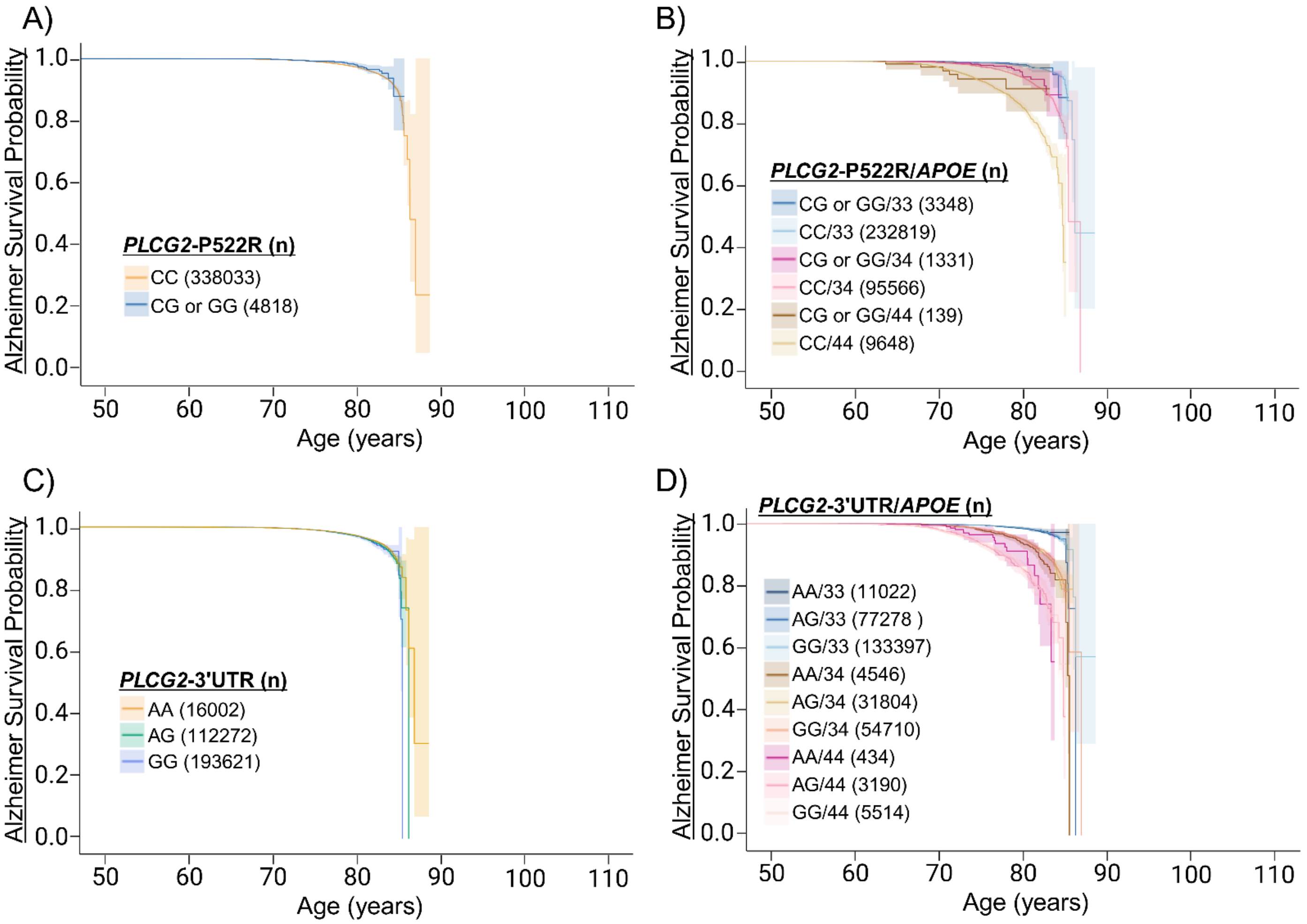

Supplementary Figure 5. *PLCG2*-P522R and *PLCG2*-3 UTR variant analyses in UK Biobank. To investigate the impact of the variants on AD onset, Kaplan-Meier curves on UK Biobank endpoint data were utilized, with the focus on *APOE* ε3- and *APOE* ε4-carrying Caucasian individuals >50 years of age. The curves illustrate the AD-free time in years, starting from age 50 until AD diagnosis or the end of follow-up for the controls. Shaded area indicates 95% confidence interval. The AD onset by A) *PLCG2*-P522R, B) *PLCG2*-P522R stratified with *APOE,* C) *PLCG2*-3'UTR, and D) *PLCG2*-3'UTR stratified with *APOE*. *APOE*: ε3/ε3=33, ε3/ε4=34 and ε4/ε4=44.

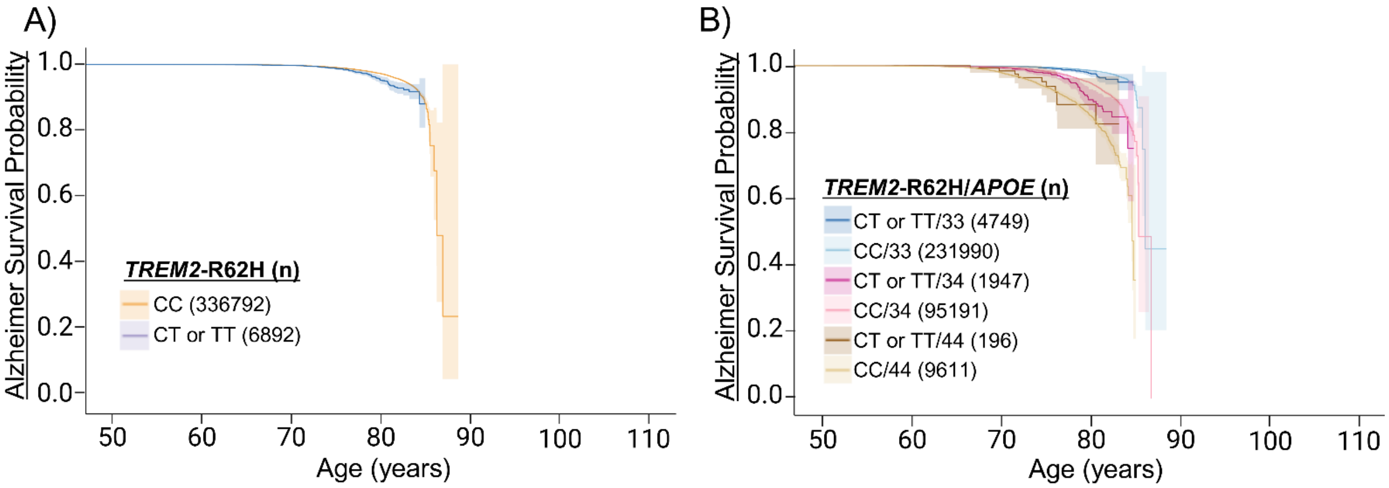

Supplementary Figure 6. *TREM2*-R62H variant analyses in UK Biobank. To investigate the impact of the variants on AD onset, Kaplan-Meier curves on UK Biobank endpoint data were utilized, with the focus on *APOE* ε3- and *APOE* ε4-carrying Caucasian individuals >50 years of age. The curves illustrate the AD-free time in years, starting from age 50 until AD diagnosis or the end of follow-up for the controls. Shaded area indicates 95% confidence interval. The AD onset by A) *TREM2*-R62H and B) *TREM2*-R62H stratified with *APOE*. *APOE*: ε3/ε3=33, ε3/ε4=34 and ε4/ε4=44.

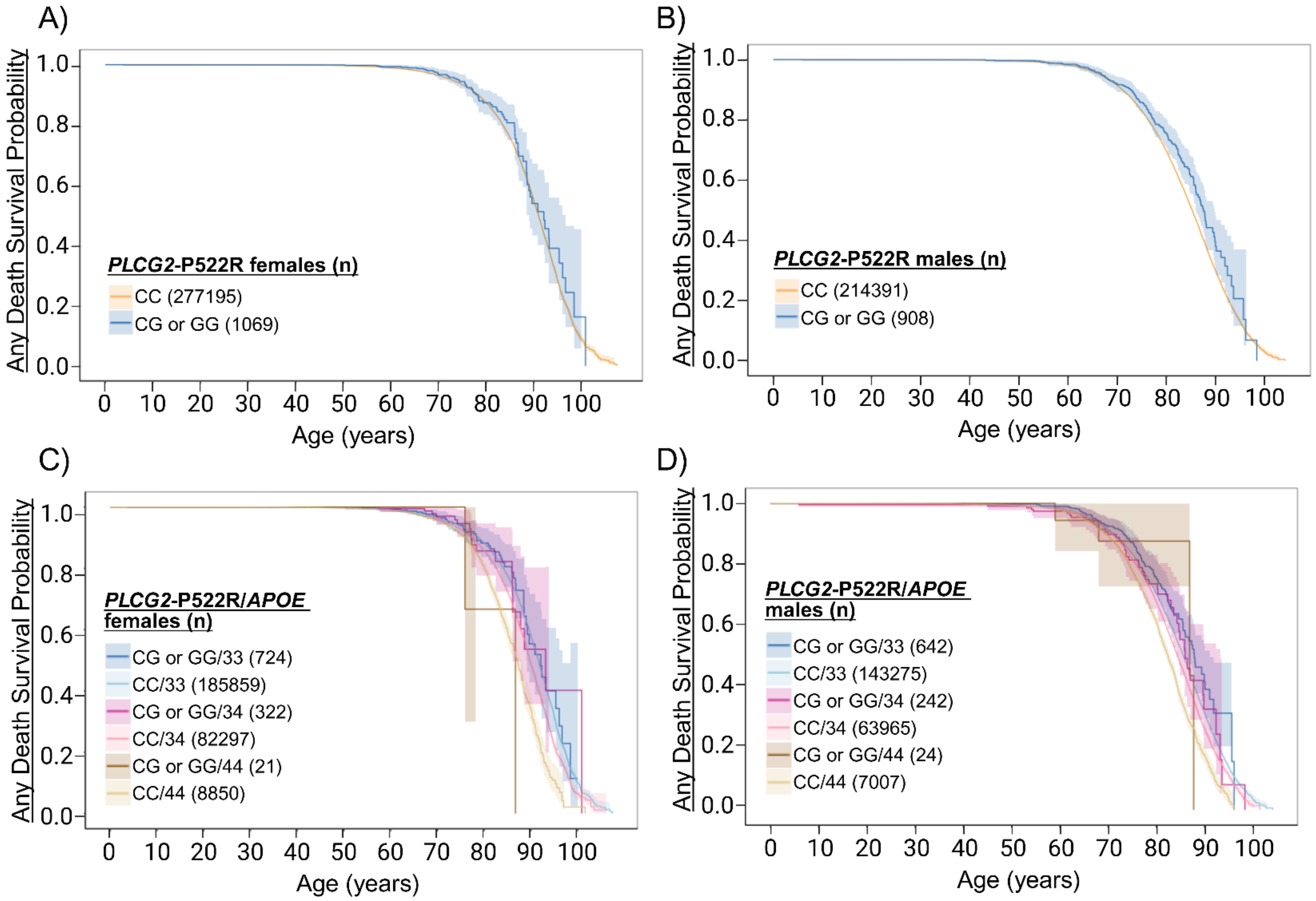

Supplementary Figure 7. *PLCG2*-P522R variant does not influence longevity. To investigate the impact of the *PLCG2*-P522R influence on longevity, Kaplan-Meier curves on FinnGen endpoint data were utilized. The curves illustrate age of death in years. Shaded area indicates 95% confidence interval. A) *PLCG2*-P522R females and B) males as well as C) females and D) males with *APOE* ε4 allele in "ANY DEATH" endpoint. *APOE*: ε3/ε3=33, ε3/ε4=34 and ε4/ε4=44.

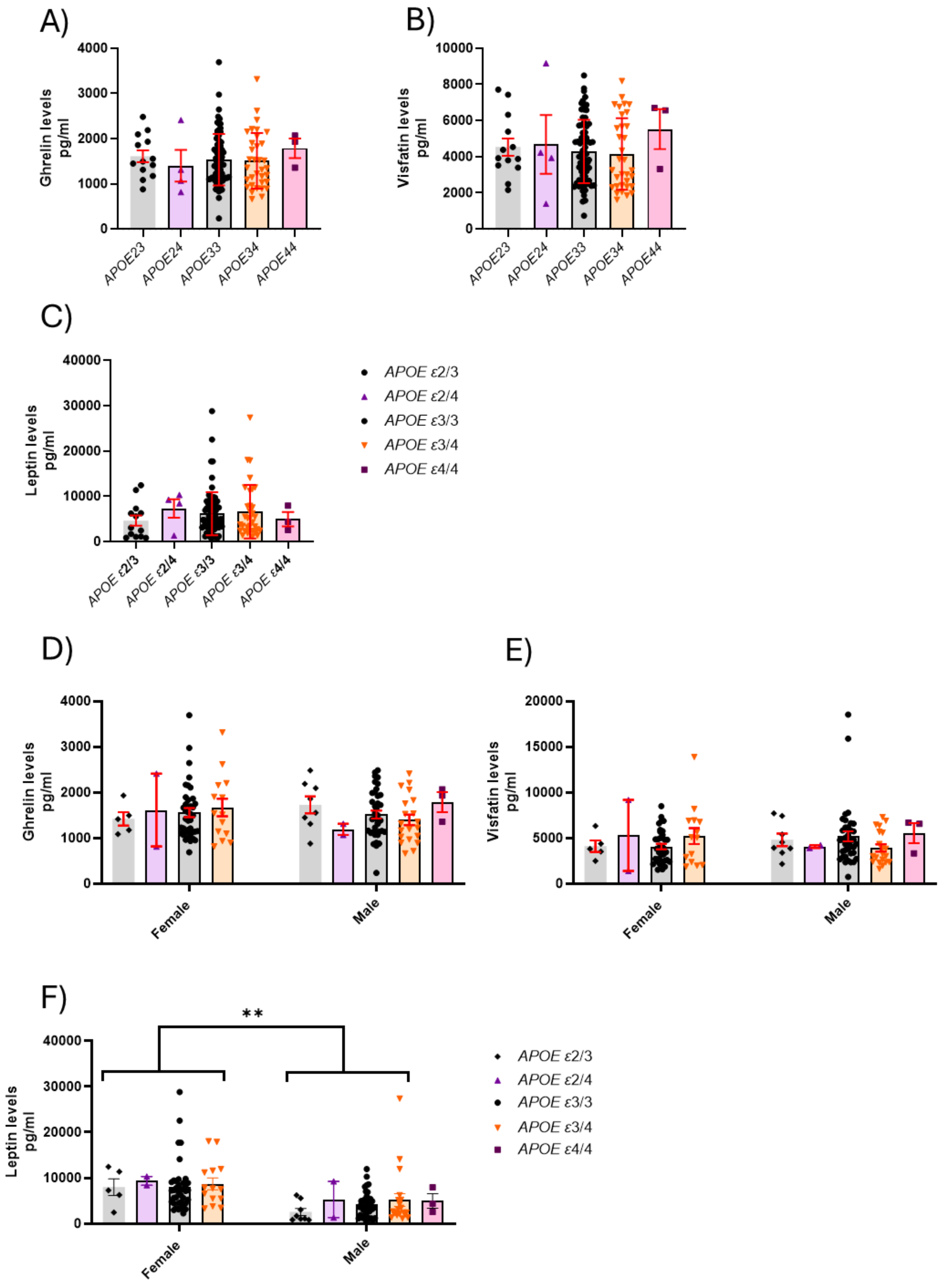
A)
Ghrelin levels pg/ml
0
1000
2000
3000
4000
APOE23
APOE24
APOE33
APOE34
APOE44
B)
Visfatin levels pg/ml
0
2000
4000
6000
8000
10000
APOE23
APOE24
APOE33
APOE34
APOE44
C)
Leptin levels pg/ml
0
10000
20000
30000
40000
APOE ε2/3
APOE ε2/4
APOE ε3/3
APOE ε3/4
APOE ε4/4
APOE ε2/3
APOE ε2/4
APOE ε3/3
APOE ε3/4
APOE ε4/4
D)
Ghrelin levels pg/ml
0
1000
2000
3000
4000
Female
Male
E)
Visfatin levels pg/ml
0
5000
10000
15000
20000
Female
Male
F)
**
Leptin levels pg/ml
0
10000
20000
30000
40000
Female
Male
APOE ε2/3
APOE ε2/4
APOE ε3/3
APOE ε3/4
APOE ε4/4

Supplementary Figure 8. Plasma ghrelin, visfatin, and leptin levels in relation to *APOE* ε3 and *APOE* ε4 carriership. Plasma A) ghrelin, B) visfatin, and C) leptin between *APOE* ε3 and *APOE* ε4 carriers. Plasma D) ghrelin, E) visfatin, and E) leptin levels between *APOE* ε3 and *APOE* ε4 carriers and sexes. Mean ± SEM; Independent samples t test (non-parametric) or Two-way ANOVA, Tukey's, Bonferroni correction; **<0.01; n(*APOE* ε2/ε3)=5-13, n(*APOE* ε2/ε4)=2-4, n(*APOE* ε3/ε3)=38-77, n(*APOE* ε3/ε4)=14-34, and n(*APOE* ε4/ε4)=3.

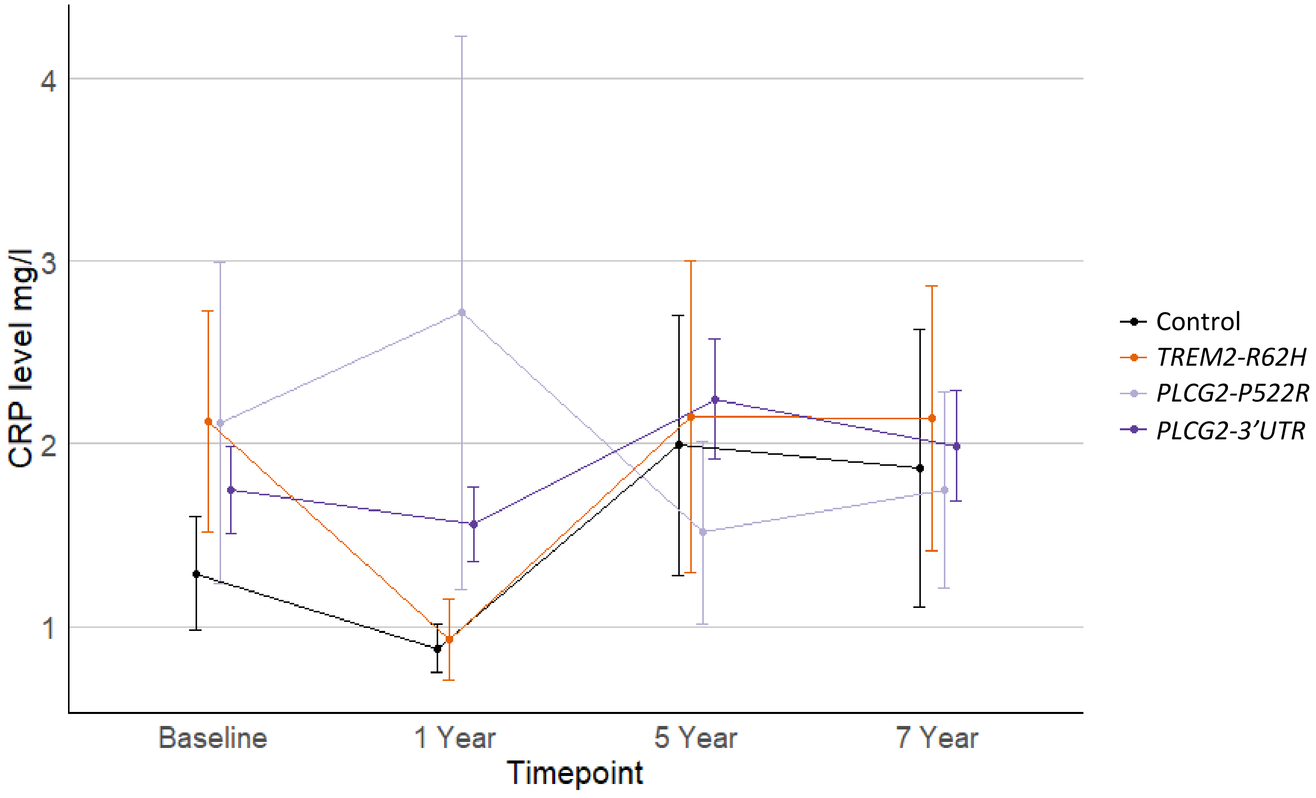

Supplementary Figure 9. Plasma CRP levels over time in individuals from the FINGER cohort at baseline, and one-, five-, and seven-year follow-up. Linear Mixed-Effects Model was used. Mean±SEM. n(control)=24-55, n(P522R)=4-6, n(R62H)=11-18, and n(3'UTR)=40-59.
