# Supplementary material for: Protective PLCG2 variants associate with a delayed onset of Alzheimer’s disease among heterozygous APOE ε4 carriers
Source: Alzheimers Res Ther. 2026 Jan 31;18:53. doi: 10.1186/s13195-026-01957-1 (PMC12964913; doi:10.1186/s13195-026-01957-1)
Supplement: Supplementary file 3 — Supplementary Material 3. Supplementary Methods [file 13195_2026_1957_MOESM3_ESM.pdf]

UK Biobank methods

Cohort

UK Biobank (UKB) is a multi-centre cohort study of approximately 502,000 participants who were recruited at the age of 39–73 years in England, Scotland, and Wales between 2006 and 2010 [1]. In this study, we included Caucasian participants who had passed sample-level genotyping quality control (genetic/phenotypic sex mismatches, excess heterozygosity, aneuploidy), had not withdrawn consent, were older than 50 years, and had APOE ε3/3, ε3/4, and ε4/4 genotypes (n = 343,684). Specific n-number per different genotype groups can be found from Supplementary Figures 5-6.

Genotyping methods

All UKB study subjects (Supp. Table 3.) have undergone genome-wide genotyping. Most of the subjects have been genotyped using the UK Biobank Axiom array by Affymetrix (now part of ThermoFisher Scientific). Approximately 50,000 subjects have been genotyped using the UK BiLEVE Axiom array. Quality controlled genotype data has been imputed using Haplotype Reference Consortium and UK10K haplotype resources to generate genotypes for approximately 96 million variants. All genotype data are in the human genome build GRCh37/hg19.

UKB phenotypes

AD was ascertained through linked electronic health records (EHR) and death records available up to July 2024, defining as cases those who had ICD-10 code in the G30 category, or primary care codes as previously used (https://www.medrxiv.org/content/10.1101/2023.11.16.23298475v6). The remaining individuals were considered as controls.

Statistical analysis

Statistical analyses were conducted as in FinnGen with the exception that survimer (v0.5.1) package version was different. Mean age of the UKB AD cases was 75.1±5.7 and controls 70.7±7.9 years.

References:

[1] Bycroft C, Freeman C, Petkova D, Band G, Elliott LT, Sharp K, et al. The UK Biobank resource with deep phenotyping and genomic data. Nature. 2018;562:203-9.
