# Supplementary material for: Protective PLCG2 variants associate with a delayed onset of Alzheimer’s disease among heterozygous APOE ε4 carriers
Source: Alzheimers Res Ther. 2026 Jan 31;18:53. doi: 10.1186/s13195-026-01957-1 (PMC12964913; doi:10.1186/s13195-026-01957-1)
Supplement: Supplementary file 4 — Supplementary Material 4. Supplementary Tables [file 13195_2026_1957_MOESM4_ESM.pdf]

Supplementary Table 1. The *PLCG2*-3'UTR cox regression model adjusted by sex in FinnGen.

	Hazard ratio (HR)	95% Confidence interval: lower-upper	p value
PLCG2-3'UTR heterozygous	0.8986	0.8462-0.9543	0.000497
PLCG2-3'UTR homozygous	0.8438	0.7950-0.8956	$2.35x10^{-8}$
SEXmale	1.0909	1.0535-1.1296	$9.87x10^{-7}$

Global Schoenfeld residual p=0.12

Supplementary Table 2. The *TREM2*-R62H cox regression model adjusted by sex in FinnGen.

	Hazard ratio (HR)	95% Confidence interval: lower-upper	p value
TREM2-R62H	1.091	0.9163-1.249	0.204
SEXmale	1.091	1.0536-1.130	$9.71x10^{-7}$

Global Schoenfeld residual p=0.19

Supplementary Table 3. Summary statistics of UK biobank cohort

Total (n)	UK biobank cohort
Study subjects	486745
Mean age	70.3±8.0
Females/Males	263967/222778
APOE ε4 carriers	126137
PLCG2-P522R	6337
PLCG2-3’UTR	431218
TREM2-R62H	9290
AD cases	4742

n=total number

Supplementary Table 4. The *PLCG2*-P522R cox regression model adjusted by sex in UK biobank

	Hazard ratio (HR)	95% Confidence interval: lower-upper	p value
PLCG2-P522R	0.7854	0.5815-1.061	0.115
SEXmale	1.0052	0.9434- 1.071	0.872

Global Schoenfeld residual p=0.85

Supplementary Table 5. The *PLCG2*-3'UTR cox regression model adjusted by sex in UK biobank

	Hazard ratio (HR)	95% Confidence interval: lower-upper	p value
PLCG2-3'UTR heterozygous	0.9919	0.8507- 1.157	0.918
PLCG2-3'UTR homozygous	0.9318	0.8020- 1.083	0.356
SEXmale	0.9943	0.9312- 1.062	0.865

Global Schoenfeld residual p=0.076

Supplementary Table 6. The *PLCG2*-P522R *APOE* stratified cox regression model adjusted by sex in UK biobank. Different *APOE* groups were analyzed separately.

	Hazard ratio (HR)	95% Confidence interval: lower-upper	p value
PLCG2-P522R/APOE33	0.9160	0.5677-1.478	0.719
SEXmale/APOE33	1.2573	1.1289-1.400	3.09×10^{-5}
PLCG2-P522R/APOE34	0.7026	0.4522-1.0916	0.116365
SEXmale/APOE34	0.8549	0.7813-0.9355	0.000646
PLCG2-P522R/APOE44	0.6644	0.2972-1.485	0.319
SEXmale/APOE44	0.9496	0.8058-1.119	0.537

Global Schoenfeld residuals: APOE33 p=0.42, APOE34 p=0.37, APOE44 p=0.13

Supplementary Table 7. The *PLCG2*-3'UTR *APOE* stratified cox regression model adjusted by sex in UK biobank. Different *APOE* groups were analyzed separately.

	Hazard ratio (HR)	95% Confidence interval: lower-upper	p value
PLCG2-3'UTR heterozygous/APOE33	1.1333	0.8625-1.489	0.369034
PLCG2-3'UTR homozygous/APOE33	1.0100	0.7728-1.320	0.942450
SEXmale/APOE33	1.2331	1.1029-1.379	0.000234
PLCG2-3'UTR heterozygous/APOE34	0.8305	0.6783-1.0168	0.072103
PLCG2-3'UTR homozygous/APOE34	0.8066	0.6628-0.9817	0.032010
SEXmale/APOE34	0.8476	0.7724-0.9302	0.000491
PLCG2-3'UTR heterozygous/APOE44	1.3603	0.8393-2.205	0.212
PLCG2-3'UTR homozygous/APOE44	1.3555	0.8431-2.179	0.209
SEXmale/APOE44	0.9595	0.8096-1.137	0.634

Global Schoenfeld residuals: APOE33 p=016., APOE34 p=0.12, APOE44 p=0.132

Supplementary Table 8. The *TREM2*-R62H cox regression model adjusted by sex in UK biobank

	Hazard ratio (HR)	95% Confidence interval: lower-upper	p value
TREM2-R62H	1.4366	1.1832- 1.747	0.00026
SEXmale	1.0026	0.9427- 1.070	0. 89116

Global Schoenfeld residual p=0.88

Supplementary Table 9. The TREM2-R62H *APOE* stratified cox regression model adjusted by sex in UK biobank. Different *APOE* groups were analyzed separately.

	Hazard ratio (HR)	95% Confidence interval: lower-upper	p value
TREM2-R62H/APOE33	1.5270	1.106-2.108	0.0101
SEXmale/APOE33	1.2557	1.128-1.398	$3.4x10^{-5}$
TREM2-R62H /APOE34	1.5595	1.1955-2.0344	0.001051
SEXmale/APOE34	0.8541	0.7806-0.9345	0.000592
TREM2-R62H /APOE44	0.8712	0.4660-1.629	0.666
SEXmale/APOE44	0.9506	0.8069-1.120	0.545

Global Schoenfeld residuals: APOE33 p=0.55, APOE34 p=0.41, APOE44 p=0.40

Supplementary Table 10. Comparison of plasma ghrelin levels between genotypes with age, sex, or *APOE ε4* status as co-variates in the FINGER cohort. Multilinear regression model was used.

	Unstandardized Coefficients		*Standardized Coefficients*	*t*	*Sig. (p)*	*95,0% Confidence Interval for B*	
	B	*Std. Error*	*Beta*			*Lower Bound*	*Upper Bound*
(Constant)	*3,373*	*0,291*		*11,576*	*0,000*	*2,793*	*3,953*
PLCG2 P522R seq.16.81942028.C.G	*0,227*	*0,079*	*0,312*	*2,862*	*0,005*	*0,069*	*0,385*
*APOE ε*4 status	*0,003*	*0,042*	*0,008*	*0,076*	*0,940*	*-0,080*	*0,086*
Age	*-0,003*	*0,004*	*-0,090*	*-0,815*	*0,418*	*-0,012*	*0,005*
Sex	*0,009*	*0,038*	*0,026*	*0,242*	*0,809*	*-0,067*	*0,086*

Dependent variable: logarithmic ghrelin levels

Supplementary Table 11. Effect of *PLCG2*-P522R variant on plasma visfatin levels between genotypes with age, sex, or *APOE ε4* status as co-variates in the FINGER cohort. Multilinear regression model was used.

	Unstandardized Coefficients		*Standardized Coefficients*	*t*	*Sig. (p)*	*95,0% Confidence Interval for B*	
	B	*Std. Error*	*Beta*			*Lower Bound*	*Upper Bound*
(Constant)	*4,021*	*0,385*		*10,454*	*0,000*	*3,255*	*4,787*
PLCG2 P522R seq.16.81942028.C.G	*0,198*	*0,105*	*0,212*	*1,894*	*0,062*	*-0,010*	*0,407*
*APOE ε*4status	*-0,015*	*0,055*	*-0,031*	*-0,274*	*0,785*	*-0,125*	*0,095*
Age	*-0,005*	*0,005*	*-0,107*	*-0,945*	*0,348*	*-0,016*	*0,006*
Sex	*0,009*	*0,038*	*0,026*	*0,242*	*0,809*	*-0,067*	*0,086*

Dependent variable: logarithmic visfatin levels

Supplementary Table 12. Effect of *PLCG2*-P522R on plasma leptin levels between genotypes with age, sex, or *APOE ε4* status as co-variates in the FINGER cohort. Multilinear regression model was used.

	Unstandardized Coefficients		*Standardized Coefficients*	*t*	*Sig. (p)*	*95,0% Confidence Interval for B*	
	B	*Std. Error*	*Beta*			*Lower Bound*	*Upper Bound*
(Constant)	*2,714*	*0,515*		*5,271*	*0,000*	*1,689*	*3,739*
PLCG2 P522R seq.16.81942028.C.G	*-0,100*	*0,140*	*-0,065*	*-0,716*	*0,476*	*-0,380*	*0,179*
APOE ε4status	*0,110*	*0,074*	*0,135*	*1,488*	*0,141*	*-0,037*	*0,257*
Age	*0,004*	*0,007*	*0,045*	*0,500*	*0,618*	*-0,011*	*0,018*
Sex	*0,467*	*0,068*	*0,621*	*6,870*	*0,000*	*0,332*	*0,602*

Dependent variable: logarithmic leptin levels
